# 4-Aminopyridine induced hyperpolarizing oscillations in pediatric human epileptic tissue are network-driven potassium currents that are abolished by activation of KCNQ2–5 (Kv7.2-Kv7.5) channels

**DOI:** 10.1016/j.nbd.2025.107252

**Published:** 2025-12-26

**Authors:** J. Keenan Kushner, Paige B. Hoffman, Christine R. Brzezinski, Brent R. O’Neill, Todd C. Hankinson, Charles C. Wilkinson, Michael H. Handler, Charles A. Hoeffer, Allyson L. Alexander

**Affiliations:** a Department of Neurosurgery, School of Medicine, University of Colorado | Anschutz Medical Campus, Aurora, CO 80045, USA; b Neuroscience Graduate Program, University of Colorado, Anschutz Medical Campus, Aurora, CO 80045, USA; c Division of Pediatric Neurosurgery, Children’s Hospital Colorado, Aurora, CO 80045, USA; d Institute for Behavioral Genetics, University of Colorado, Boulder, CO 80309, USA; e Department of Integrative Physiology, University of Colorado, Boulder, CO 80309, USA; f Linda Crnic Institute, University of Colorado, Anschutz Medical Campus, Aurora, CO 80045, USA

**Keywords:** Drug-resistant epilepsy, Pediatric epilepsy, Human brain tissue, Network oscillations, M-current, GABA

## Abstract

Epilepsy is one of the most common neurological disorders worldwide. Despite the availability of many anti-seizure medicines (ASMs), about 30 % of patients with epilepsy develop drug-resistant epilepsy. Unfortunately, the mechanisms of ictogenesis in patients with drug-resistant epilepsy remain to be elucidated. Here, we used 4-aminopyridine (4-AP) to study interictal-like oscillations in human epileptic neocortex. 4-AP is a voltage-gated potassium channel blocker commonly used to induce seizure-like activity in ex vivo brain slices. We observed that 4-AP induced neuronal bursting and robust slow, hyperpolarizing oscillations (HypOs) in layer 2/3 (L2/3) pyramidal neurons (PNs). Using paired recordings, we demonstrate that neuronal bursting and HypOs are synchronized between neighboring L2/3 PNs. We also determined that 4-AP-induced HypOs are potassium currents that were not mediated by GABA_A_/_B_ receptors, NMDA receptors or AMPA receptors, or NKCC1 and KCC2 channels. Instead, HypOs are dependent on network activity and are impacted by gap junction blockade. Interestingly, HypOs were eliminated by activation, but not inactivation, of KCNQ2–5 (Kv7.2-Kv7.5) channels and were reduced via intercellular calcium chelation suggesting a role for calcium in KCNQ channel activation. Our results indicate 4-AP-induced HypOs are due to GABAergic interneuron synchronization, which leads to local extracellular potassium fluctuations without the need for GABA neurotransmission. Moreover, KCNQ2–5 channel activation can help stabilize potassium fluctuations, resulting in cessation of interictal-like events.

## 1. Introduction

Childhood epilepsy is a common disorder, with a reported prevalence ranging from 0.5 %–4.4 % (Banerjee et al., 2009; Baulac et al., 2015; Camfield and Camfield, 2015; Farghaly et al., 2018; LaGrant et al., 2020; Pakdaman et al., 2021; Wanigasinghe et al., 2019; Zack and Kobau, 2017). As many as 30 % of children with epilepsy will develop drug-resistant epilepsy, leading to a significant decrease in quality of life. Although many excellent rodent models exist of drug-resistant childhood epilepsies, there are crucial differences between the circuitry and physiology of human and rodent epileptic cortex. Thereby, surgical resection in patients with drug-resistant epilepsies provides a unique opportunity to investigate and understand the human epileptic brain.

To study epileptic human tissue, a variety of ex vivo slice experimental paradigms have been implemented to induce epileptiform activity. One of these is the wash-on of 4-aminopyridine (4-AP), a voltage-gated potassium channel blocker that blocks members of the Kv1, Kv3, and Kv4 families (Abdijadid et al., 2015; Cepeda et al., 2018; Levinson et al., 2020; Ruschenschmidt et al., 2004; Simeone et al., 2011; Wulff and Zhorov, 2008). 4-AP has been shown to induce both synchronized network events (interictal-like) and seizure-like (ictal) activity in human acute slices without specifically acting on glutamatergic or GABAergic neurotransmission allowing researchers to probe into the role these neurotransmitter systems play in epileptiform activity (Avoli and Jefferys, 2016; Avoli et al., 1988). We and others have previously reported the effects of 4-AP on neuronal excitability including widening of action potential half-widths and perturbing firing rate accommodation, increasing GABAergic and glutamatergic neurotransmission, promoting neuronal bursting in excitatory and inhibitory neurons, and inducing slow, rhythmic oscillations (Abdijadid et al., 2015; Avoli et al., 1999; Cepeda et al., 2018; Kushner et al., 2025; Levinson et al., 2020; Mendez-Rodriguez et al., 2021). The 4-AP induced slow, rhythmic oscillations have been characterized as periodic, synchronized, oscillatory rises in extracellular potassium ([K+]_o_) and a drop in extracellular calcium ([Ca^2+^]_o_). The transition to ictal activity occurs following these interictal oscillations and is characterized by long-lasting rises in [K+]_o_ that occur concurrently with paroxysmal discharges (long-lasting neuronal bursts) (Avoli et al., 1995; D’Antuono et al., 2004; Kohling and Avoli, 2006). However, the mechanism underlying interictal events and how the interictal to ictal transition occurs is not yet fully understood.

The inhibitory control of epileptiform activity via GABA_A_ receptors has shown counterintuitive results, as ex vivo studies using 4-AP have found that both activation and blockade of GABA_A_ receptors can initiate or terminate ictal activity (D’Antuono et al., 2004). On the other hand, the metabotropic GABA_B_ receptor has been found to reduce ictal activity when activated and enhance ictal activity when blocked, suggesting a role in controlling ictal activity thresholds via tonic inhibition (D’Antuono et al., 2004; Levinson et al., 2020). Yet, neither GABA_A_ nor GABA_B_ activation fully suppress *interictal* activity induced by 4-AP, indicating that epileptiform network oscillations may occur without GABAergic neurotransmission.

Here, we demonstrate that 4-AP (100 μM) induces reproducible and robust interictal-like oscillations and neuronal bursts in L2/3 PNs recorded from ex vivo epileptic human cortical tissues, similar to previous results (Cepeda et al., 2018; Levinson et al., 2020). We also demonstrate that the slow, rhythmic hyperpolarizing oscillations, but not L2/3 PN bursting, occurs in non-epileptic control tissue. By investigating the mechanism by which 4-AP generates these oscillations we establish that 1) 4-AP causes synchronized bursting and synchronized hyperpolarizing oscillations via paired recordings, 2) these hyperpolarizing oscillations are dependent on network activity and are not mediated by GABA_A_/_B_ receptors, 3) potassium is the main ionic component of this hyperpolarization, 4) HypOs are partially mediated by GABAergic interneuron synchronization via gap junctions, and 5) HypOs are eliminated by KCNQ2–5 (Kv7.2-Kv7.5) channel activation.

## 2. Materials and methods

### 2.1. Acute human brain slice preparation for electrophysiology

Prior to initiating our study, we obtained approval from the Colorado Multiple Institutional Review Board and Children’s Hospital Colorado. Informed consent was obtained from the patient or their guardian. Eligible subjects for the medically refractory epilepsy group included patients undergoing resection of epileptogenic tissue at our pediatric hospital. For all epilepsy patients, the resected neocortical tissue used for recordings was taken from the epileptogenic zone (seizure focus). Eligible control patients included patients undergoing resection of a deep-seated tumor or vascular malformation, in whom removal of “normal” overlying cortex was required to surgically access the lesion, and no additional tissue was taken beyond that which was clinically necessary. Patients were excluded from the control arm if they had any known history of seizures. The age, sex, location of resected tissue, and surgical pathology for each patient are reported in Table 1. Patients ranged in age from 1 to 21 years. All surgeries were performed at Children’s Hospital Colorado by authors ALA, TCH, BRO, CCW, or MHH.

Immediately following removal of cortical tissue from the operative bed, the cortical tissue was submerged in 0–4 °C carbogenated (95 % O_2_–5 % CO_2_) *N*-methyl-D-glucamine (NMDG)-substituted artificial cerebrospinal fluid (ACSF) (Ting, 2018). Composition of NMDG ACSF is as follows ((in mM): 92 NMDG, 2.5 KCl, 1.25 NaH_2_PO_4_, 30 NaHCO_3_, 20 4-(2-hydroxyethyl)-1-pip-erazineethanesulfonic acid (HEPES), 25 d-glucose, 2 thiourea, 5 Na-ascorbate, 3 Na-pyruvate, 0.5 CaCl_2_·4H_2_O and 10 MgSO_4_·7H_2_O, pH adjusted to 7.3–7.4 with concentrated hydrochloric acid, osmolality~305mOsm/kg). The tissue was then transported to the laboratory, under continuous bubbling with carbogen. The total duration from operating room to the initiation of brain slicing was 15–20 min. The tissue was then placed in a petri dish containing carbogenated NMDG ACSF at 0–4 °C. The tissue was then cut into blocks of approximately 1cm^3^ with a scalpel blade if the resected portion was large. The arachnoid membrane and surface cortical vessels were carefully removed. No effort was made to remove the pia mater due to the risk of damaging the underlying grey matter. The tissue block was then superglued onto a vibratome stage (Leica Biosystems) and immersed in 0–4 °C carbogenated NMDG ACSF. Acute slices (400 μm) were prepared, slicing perpendicular to the pial surface to ensure that the dendrites of large PNs were preserved. Slices were then incubated in carbogenated NMDG ACSF at ~35°C for 12 min and then transferred to a room temperate (~23°C) carbogenated modified-HEPES ACSF ((in mM): 92 NaCl, 2.5 KCl, 1.2 NaH_2_PO_4_, 30 NaHCO_3_, 20 HEPES, 25 d-glucose, 2 thiourea, 5 Na-ascorbate, 3 Na-pyruvate, 2 CaCl_2_·4H_2_O, 2 MgSO_4_·7H_2_O, pH adjusted to 7.3–7.4 with HCl, osmolality~305mOsm/kg). The slices remained in the~23°C modified-HEPES ACSF for at least 30 min before being transferred to the recording chamber for electrophysiology experiments.

### 2.2. Mouse model of malformation of cortical development (MCD)

All animal experiments were approved by the University of Colorado Anschutz Institutional Care and Use Committee. Every effort was made to minimize the number of animals. All mice were obtained from The Jackson Laboratory and were housed under a 12/12 h light/dark cycle. Food and water were available ad libitum. Analgesia and euthanasia methods were chosen under the advisement of the veterinarians at our institution.

#### 2.2.1. In utero electroporation

In utero electroporation was performed as previously described (Hoffman et al., 2023; Hsieh et al., 2016). Briefly, we performed electroporation in mouse embryonic brains on embryonic day 15.5 in timed-pregnant CD-1 dams under general anesthesia. To generate malformations of cortical development (MCD) mice, a constitutively active Rheb (Rheb^CA^) plasmid was co-electroporated with a pCAG-GFP plasmid. Controls were electroporated the pCAG-GFP plasmid only. The pCAG-Rheb^CA^ plasmid was a gift from Dr. Maehama (Maehama et al., 2008). The electroporation was aimed at the motor/somatosensory neocortex and mice were only used when the MCD was unilateral. We have previously verified that the Rheb^CA^ mice exhibit spontaneous recurrent seizures and histological features of focal cortical dysplasia (Hoffman et al., 2023).

#### 2.2.2. Acute slice preparation of mouse brains for electrophysiology

Slice electrophysiology experiments were conducted on mice aged to postnatal days (P)25–P35. Experiments were conducted on both male and female mice. Mice were exposed to a rising concentration of carbon dioxide (CO_2_) at 0.6 L/min flow rate until loss of consciousness followed by decapitation. Brains were quickly removed by dissection and glued cerebellar side-down on the vibratome stage and immersed in 0–4 °C carbogenated cutting solution ((in mM):45 sucrose, 25 d-glucose, 85 NaCl, 2.5 KCl, 1.25 NaH_2_PO_4_, 25 NaHCO_3_, 0.5 CaCl_2_, and 7 MgCl_2_, osmolality~290–300 mOsm/kg, pH adjusted to 7.3–7.4). 300 μm coronal slices containing unilateral GFP-positive neurons were kept and incubated at~35°C in carbogenated recording ACSF ((in mM: 124 NaCl, 2.5 KCl, 1.25 NaH_2_PO_4_, 25 NaHCO_3_, 10 d-glucose, 2 CaCl_2_, and 2 MgCl_2_, osmolality~290–300 mOsm/kg, pH adjusted to 7.3–7.4) for 30 min followed by 30 min at~23°C before electrophysiology recordings.

### 2.3. Electrophysiology

#### 2.3.1. Patch clamp recordings

For ex vivo human slice whole-cell patch clamp recordings, slices were placed in a submerged slice chamber and perfused at a rate of 2 mL/min with heated (~33°C) recording ACSF ((in mM): 124 NaCl, 2.5 KCl, 1.2 NaH_2_PO_4_, 24 NaHCO_3_, 5 HEPES, 12.5 d-glucose, 2 CaCl_2_·4H_2_O, 2 MgSO_4_·7H_2_O, pH adjusted to 7.3–7.4 with HCl, osmolality~305mOsm/kg). Mouse slice whole-cell electrophysiology used the recording ACSF mentioned above. Slices were visualized using a moving stage microscope (Scientifica, SliceScope Pro 2000) equipped with 4× and 40× water immersion objectives, differential interference contrast (DIC) optics, a SciCam Pro camera (Scientifica), and Micro-Manager 1.4 (Open Imaging). L2/3 PNs were targeted under DIC based on their visible apical dendrite and large, pyramidal soma and were confirmed via intrinsic property analysis as previously reported (Kushner et al., 2025). Whole-cell patch clamp recordings were performed using borosilicate glass pipettes (outer diameter: 1.5 mm; Sutter Instrument, Cat# BF150–86–10) pulled to resistances of 3–6 MΩ. Electrical recordings were acquired with a MultiClamp 700B amplifier (Devinsky et al., 2018) and were sent through a Hum Bug Noise Eliminator (Quest Scientific) to be then converted to a digital signal with the Axon^™^ Digidata^®^ 1440 A digitizer using pCLAMP 10.7 software (Devinsky et al., 2018). Data were discarded if the access resistance >25 MΩ. In current clamp mode, compensation for voltage variations was achieved using a bridge balance circuit. Data were sampled at 10 kHz. No liquid junction potential correction was performed.

#### 2.3.2. Internal solutions

Pipettes were backfilled with 3 different internal solutions to decipher ionic contribution to the 4-AP-induced HypOs. The K-gluconate (high K^+^) internal was used unless otherwise noted and consisted of (in mM): 135 K gluconate, 20 KCl, 10 HEPES,0.1 EGTA, 2 Mg-ATP, 0.3 Na_2_-GTP. The high Cl^−^ internal consisted of (in mM): 135 KCl, 10 HEPES, 20 K-gluconate, 0.1 EGTA, 2 Mg-ATP, 0.3 Na_2_-GTP. The low K^+^ internal consisted of (in mM): 110 cesium-methanesulfonate, 20 CsCl, 10 Na-phosphocreatine, 1 QX-314, 10 HEPES, 0.5 EGTA, 4 Mg-ATP, 0.4 Na_2_-GTP. In a subset of experiments, BAPTA tetrasodium salt was added to the K-gluconate internal solution at concentrations of 5 mM, 10 mM, and 20 mM. The pH of each internal solution was adjusted to 7.3–7.4 with KOH or CsOH and internal osmolality was~290mOsm/kg.

#### 2.3.3. Ramped current injection

After achieving whole-cell configuration, L2/3 PNs were recorded from rest in current clamp mode (I_hold_ = 0pA). Following a three-second baseline period, the holding current was linearly ramped from 0 to 1000pA over 2 s. A total of 4 sweeps of data were collected for each neuron, and the data were used to determine resting membrane potential (RMP), action potential (AP) threshold, and rheobase current.

#### 2.3.4. Square current injection

Following ramped current injections, we recorded the responses of L2/3 PNs to a series of square hyperpolarizing and depolarizing current injections. Before initiation of the series of current injections, the resting membrane potential of neurons was adjusted to approximately −60 mV. Each cell was subjected to two series of 600 ms square current injections: −100 to +100pA at 10pA intervals and −250 to +1000pA at 50pA intervals. Separate recordings of 1000 ms square current steps from −250 to +1000pA at 50pA intervals were used to assess frequency adaptation over time. The data collected in these experiments were used to determine active and passive membrane properties of neurons, as previously described (Kushner et al., 2025, Svalina et al., 2021).

#### 2.3.5. Extracellular drug application

After intrinsic property protocols were run, neurons were held at −55 mV in current clamp mode and 4-aminopyridine (4-AP, 100 μM) was added to the perfusate. We then noted the time when the first oscillation was detected before starting 3-min gap-free recordings. 4-AP induced oscillations took roughly 3–6 min to occur. In cases where other drugs were washed on after the start of oscillations, a single recording where at least four oscillations occurred was completed before drug wash-on began to compensate for the run-down in HypO frequency. The effect of each drug that was washed on was recorded throughout the wash-on and effects were analyzed after 9–12 min. The drugs used and their wash-on concentrations can be found in Supplementary Table 2. For paired recordings, L2/3 PNs were within 100 μm of each other. The ramp current injection and square current injection were performed before 4-AP wash-on to validate PN intrinsic properties when the potassium-gluconate internal was used.

### 2.4. Statistical analysis

Data was excluded from analysis if the ROUT outlier analysis (Q = 5 %) found outliers. For data set groups of *n* > 20, an Anderson-Darling normality test was used and for data sets with *n* ≤ 20, a Shapiro-Wilk test was used to check for normality. Data was also checked for kurtosis and skewness values. If kurtosis or skewness were greater than ±2, a data set was non-normal, and/or the SD differed more than 2× between groups, a nonparametric test was used. For unpaired data with *n* > 10 an unpaired *t*-test was used if the data was normally distributed and a MWU test was used if the data were non-normal. For unpaired, normal data with *n* < 10 or with major differences in group size, a Welch’s t-test was used and a MWU test was used if the data were non-normal. For paired data, a Wilcoxon matched-pairs signed rank test was used unless all values were zero or *n* ≤ 5, in these cases a paired t-test was used.

For comparison of 3 groups of unpaired, nonnormal data, a Kruskal-Wallis test was performed followed by Dunn’s multiple-comparisons post hoc test and the *H* (degrees of freedom) statistic was checked against the critical chi-square value. For comparisons between 3 groups of unpaired, normal data, an ANOVA test was performed followed by a Tukey HSD post hoc test.

All statistical tests were two-tailed. Experimental numbers are reported as *n* = *x*, *y*, where *x* is the number of neurons and *y* is the number of patients. Data are presented as the mean ± SD unless otherwise stated as standard error of the mean (SEM). Statistical significance is notated in figures as the number of asterisks with the corresponding *p*-values: *p < 0.05, **p < 0.01, ***p < 0.001, ****p < 0.0001. All data analysis was performed using Clampfit or GraphPad Prism. Data visualizations were created in GraphPad Prism 9.3.1 and Adobe Illustrator.

## 3. Results

### 3.1. 4-AP-induced synchronized neuronal bursting and hyperpolarizing oscillations (HypOs)

In this study, we investigated oscillations induced by wash-on of 4-AP (100 μM). We obtained tissue from a total of 46 patients (40 with epilepsy, 6 control). The mean age was 8.6 ± 5.9 years in the epilepsy group and 8.7 ± 4.03 years in the control group. The ages ranged from 1 to 21 years in the epilepsy group and 1–12 years in the control group. In the epilepsy group, 50 % (20/40) were male, in the control group, 67 % (4/6) were male. Details of patient demographics, diagnoses, and resected cortical area are presented in Table 1. Additional clinical information is presented in Supplementary Table 1.

Previous research using local field potentials and K^+^-sensing electrodes has indicated that 4-AP-induced ictal and interictal-like activity in ex vivo epileptic human brain slices are accompanied by fluctuations of extracellular potassium (Kohling and Avoli, 2006). In whole-cell recordings, 4-AP-induced ictal-like activity is characterized by long-lasting neuronal bursts, also referred to as paroxysmal discharges. Interictal activity is thought to be mediated by slow membrane oscillations, which we reported in our previous study and has also been reported by others (Cepeda et al., 2018; Kushner et al., 2025; Levinson et al., 2020). However, the mechanisms behind these membrane oscillations and the transition to bursting have not been fully elucidated.

**Fig. 1.**
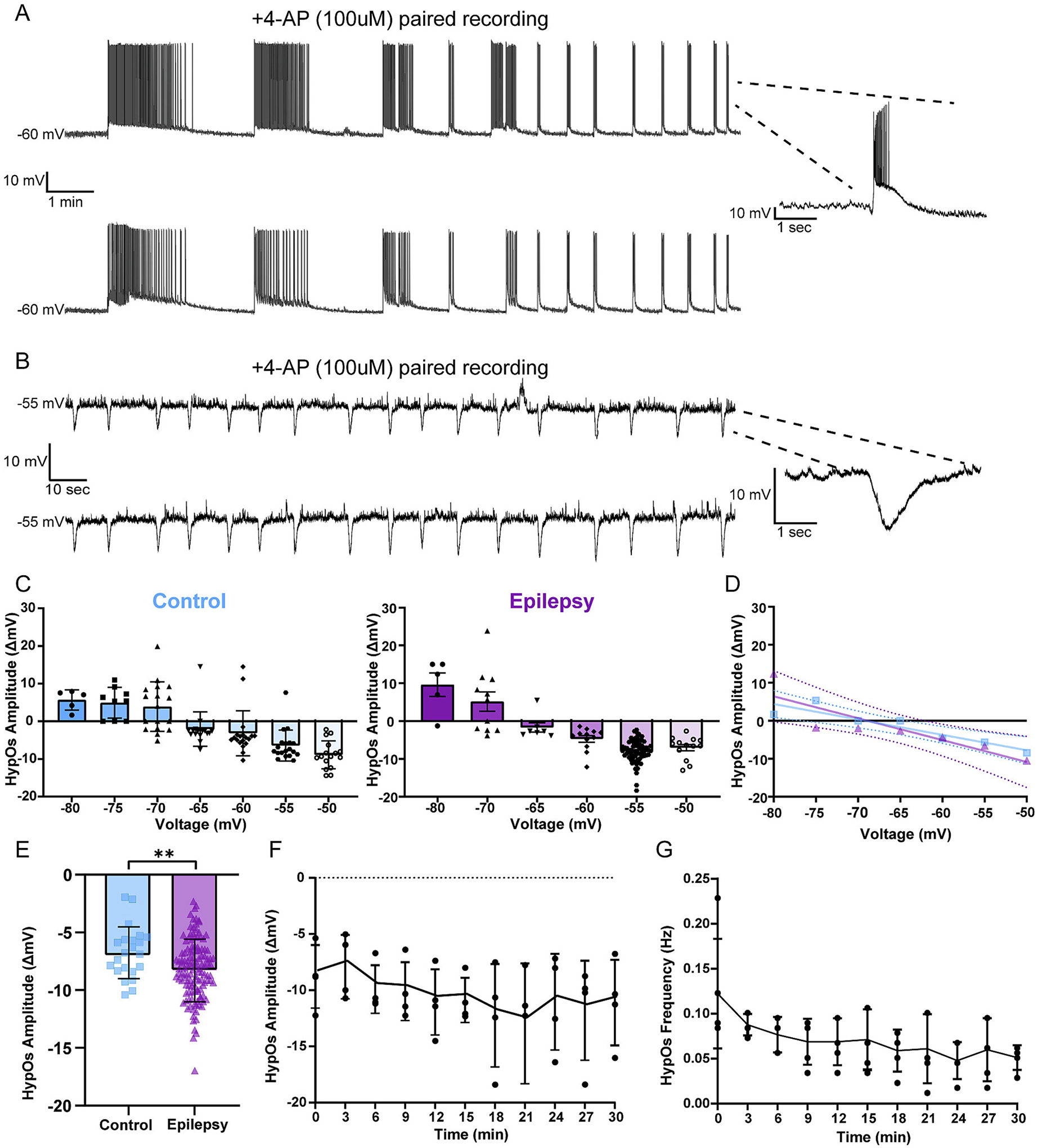
4-AP induces synchronized bursting and causes HypOs. A) Traces of paired current clamp recordings of L2/3 PNs indicating synchronized bursting after 20-min of 4-AP (100 μM) wash-on (inset: expanded individual burst). B) Traces of a paired recording of L2/3 PNs showing synchronized HypOs after 4-AP wash-on (*n* = 5) (inset: expanded individual HypO). C) The amplitude of HypOs is dependent on membrane potential, and this dependence is similar in control (left) and epileptic (right) tissue. D) Reversal potential for HypOs calculated by simple linear regression is not different between control (blue) and epileptic (purple) L2/3 PNs(*p*_*slope*_ = 0.325, *p*_*intercept*_ = 0.756). E) When L2/3 PNs were held at −55 mV, epileptic HypOs had significantly larger amplitudes compared to control HypOs (Welch’s *t*-test: *p* = 0.0020, n_control_ = 24, n_epilepsy_ = 128). F) When maintained in 4-AP alone, epileptic HypO amplitude increased over 30-min. G) Epileptic HypO frequency decreased over 30-min.

Interestingly, we only observed prolonged, repetitive bursting in a few epileptic L2/3 PNs (*n* = 5/128 epileptic) and no bursting in control L2/3 PNs (*n* = 0/25 control) in the presence of 4-AP (100 μM) alone when L2/3 PNs were held at −55 mV. We also found that this prolonged bursting activity was synchronized between neurons after 20 min of 4-AP wash-on (*n* = 1 pair; Fig. 1A). Contrary to the low percentage of bursting neurons, we reliably observed HypOs in every recorded neuron exposed to 4-AP from both epileptic and control L2/3 PNs starting regularly 3–6 min after wash-on. These HypOs were slow (1-s long), rhythmic (~0.1 Hz), and synchronized between L2/3 PNs, suggesting a network level oscillation (n = 5 pairs; Fig. 1B).

Given that epilepsy is a disorder of hyperexcitability, we were surprised by the robust and reproducible presence of synchronous *hyperpolarizing* oscillations at −55 mV. As a first step in uncovering the mechanism behind this network activity, we investigated the reversal potential of L2/3 control and epileptic HypOs (Fig. 1C). We used a simple linear regression to determine the average reversal potentials for control and epileptic L2/3 PNs (Table 2). There was no significant difference between control and epileptic reversal potential slopes (control: −69.13 mV (95 % CI [−76.61 to −64.24 mV]); epileptic: −68.83 mV (95 % CI [−81.15 to −62.07 mV]; F (1,10) = 1.071, *p*_*slope*_ = 0.325) or y-intercepts (control = −28.06 epileptic = −39.66, *p*_*intercept*_ = 0.756) indicating 4-AP-induced HypOs have similar ionic driving forces in these two groups (Fig. 1D). Importantly, the HypO reversal potential of approximately −69 mV lies between the reversal potential for chloride (E_Cl_ = −49.48 mV) and that for potassium (E_K_ = −108.87 mV) in our experimental conditions suggesting that both ions may play a role in generating the HypOs. Additionally, when comparing HypO amplitudes at −55 mV, we found that HypOs had significantly higher amplitude in epileptic (−8.26 ± 2.49 mV) compared to control L2/3 PNs (−6.52 ± 2.30 mV) L2/3 PNs (Welch’s *t*-test: *p* = 0.0020, n_control_ = 24, n_epilepsy_ = 127) (Fig. 1E). This suggests that the epileptic L2/3 circuits exhibit increased network synchrony. We compared 4-AP induced HypO amplitudes between congenital malformations of cortical development (MCD) epilepsy cases (*n* = 51, 13), patients with other epilepsies (OE, *n* = 76, 25), and control (*n* = 24, 6). We found that MCD tissue and OE cases had significantly larger HypOs compared to control cases (Control: −6.52 ± 2.30 mV, MCD: −8.91 ± 2.18 mV, OE:−7.77 ± 2.27 mV; ANOVA (0.212 (2, 148), *p* = 0.0001); Tukey post hoc tests: Control vs MCD: *p* < 0.0001; Control vs OE: *p* = 0.049). Moreover, we found that MCD HypOs were significantly larger than OE HypOs (Tukey post hoc test: MCD vs OE: *p* = 0.015) suggesting that epilepsy etiology dictates interictal-like responses induced by 4-AP. We also note that the amplitudes of epileptic HypOs increased by 26.4 % over 30 min (−8.790 ± 2.811 mV at 0 min, −11.109 ± 3.812 mV at 30 min; *n* = 4; Fig. 1F). In contrast, we observed a run-down in epileptic HypO frequency with a 37.2 % decrease over 30 min (initial frequency: 0.131 ± 0.067 Hz; final frequency: 0.049 ± 0.015 Hz; n = 4; Fig. 1G).

### 3.2. HypOs are larger in epileptic versus control tissue in a mouse model of epilepsy

To compare epileptic and control tissue with less variability than the human specimens, and to compare epileptic specimens with true controls, we recorded HypOs in a mouse model of epilepsy. We create an epileptogenic malformation of cortical development (MCD) in motor/somatosensory neocortex via focal upregulation of Rheb, which causes an upregulation of mTORC1 signaling and a lesion consisted with focal cortical dysplasia (FCD) (Gong et al., 2015; Hoffman et al., 2023; Hsieh et al., 2016). In this model, we recorded from Rheb-upregulated neurons in Layer 2/3 of primary motor cortex. Our results in mice were similar to those in the human tissue. 4-AP induced HypOs in every L2/3 PN recorded from both control and MCD mice. MCD L2/3 PNs had significantly larger HypO amplitudes compared to control L2/3 PNs (recorded at −55 mV; control: −5.37 ± 1.81 mV; MCD: −7.95 ± 3.10 mV; unpaired *t*-test: *p* = 0.014, n_control_ = 12, 6, n_MCD_ = 19, 6) (Supplementary Fig. 1B). Given that HypO amplitude is larger in epileptic versus control tissues in both human and mouse tissue, we believe that network hyperexcitability plays a leading role in amplifying HypOs.

### 3.3. HypOs are network events but are not primarily mediated by glutamate or GABA

**Fig. 2.**
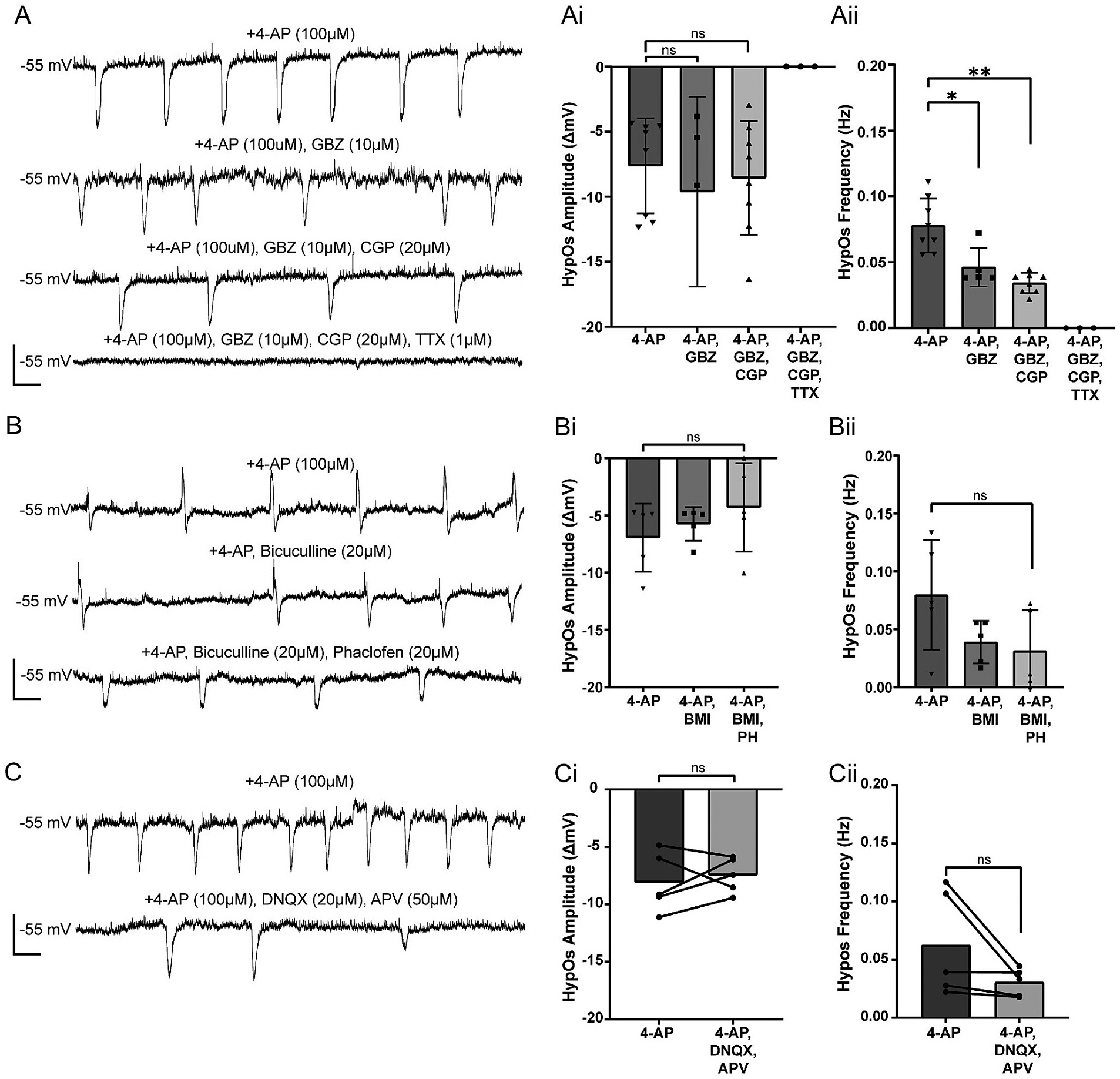
HypOs are not regulated by GABA or glutamate receptor activation. A) Example traces of 4-AP + GABA_A_ receptor blocker, gabazine (GBZ, 10 μM), GABA_B_ receptor blocker, CGP (20 μM), and Na^+^ channel blocker, tetrodotoxin (TTX, 1 μM) influence on HypOs. Ai) HypO amplitude was not significantly reduced by GBZ and CGP (Wilcoxon test: *p* = 0.250, *n* = 8), but HypOs were eliminated by consecutive addition of TTX (*n* = 3). Aii) HypO frequency was significantly reduced with wash-on of GBZ and CGP (Wilcoxon test: *p* = 0.004, n = 8). B) Example traces showing the effects of a GABA_A_ receptor blocker, bicuculline methiodide (BMI, 20 μM), and a GABA_B_ receptor blocker, Phaclofen (PH, 20 μM). These traces also demonstrate biphasic HypO with depolarizing followed by hyperpolarizing phases. BMI eliminated the depolarizing phase of the oscillations. Bi-ii) GABAergic receptor blockade with BMI and PH had no significant influence on HypO amplitude or frequency (paired *t*-test: amplitude: *p* = 0.181, frequency: *p* = 0.169, n = 5,). C) Example traces of HypOs in 4-AP alone or in the presence of glutamate receptor blockers (AMPA receptor blocker DNQX (20 μM) and NMDA receptor blocker APV (50 μM)). Ci-ii) Blockade of glutamate receptor blockers did not affect HypO amplitude or frequency (paired *t* -test: amplitude: *p* = 0.581, frequency: *p* = 0.131, n = 5). Scale bars: 10 mV, 5 s.

Since HypOs are hyperpolarizing, synchronized, have slow kinetics, and do not correlate with the reversal potential of a single ion (Avoli and Olivier, 1989), we hypothesized that a combination of GABA_A_ and GABA_B_ receptor activity might mediate human epileptic 4-AP induced HypOs. Additionally, a previous study has reported that similar 4-AP-induced oscillations are blocked with phaclofen, a GABA_B_ receptor antagonist (Levinson et al., 2020). To look at this, we held L2/3 epileptic PNs at −55 mV to record HypOs that may also have biphasic depolarizing potentials most likely mediated by GABA_A_ receptors due to our K-gluconate internal chloride reversal potential (E_Cl_ = −49.48 mV). In our hands, neither the addition of a GABA_A_R antagonist, gabazine (GBZ, 10 μM) nor a GABA_B_R antagonist, CGP35348 (CGP, 20 μM) changed the amplitude of HypOs (4-AP: −7.62 ± 3.65 mV, *n* = 8; 4-AP/GBZ: −8.88 ± 6.54 mV, *n* = 5; paired *t*-test: *p* = 0.262; 4-AP/GBZ/CGP −8.57 ± 4.38 mV, n = 8; Wilcoxon test: *p* = 0.250; Fig. 2A). However, these GABA modulators led to a significant reduction in HypO frequency (4-AP: 0.078 ± 0.02 Hz, n = 8; 4-AP/GBZ: 0.05 ± 0.01 Hz, n = 5; paired t-test *p* = 0.013; 4-AP/GBZ/CGP: 0.03 ± 0.01 Hz, n = 8; Wilcoxon test: *p* = 0.004). Although these oscillations were not abolished in the absence of GABAergic neurotransmission, some L2/3 PNs transitioned from exhibiting HypOs only to a combination of bursting and HypOs (*n* = 1/10) or bursting only (*n* = 2/10) (Supplementary Fig. 2). This suggests that GABA gates the transition from hyperpolarizing oscillations to ictal-like hyperexcitability, as previously observed. Additionally, when the sodium channel blocker tetrodotoxin (TTX, 1 μM) was consequently added, all HypOs were abolished (*n* = 3; paired *t*-test: *p* = 0.025) (Fig. 2A). This supports our assumption that HypOs are network level events and not just occurring in single cells (Fig. 2A–2Aii). In separate recordings, TTX (1 μM) was added alone after 4-AP wash-on. In these recordings, TTX completely abolished the HypOs (*n* = 4).

Given that these results were unexpected based on previous work and the slow kinetics of the HypOs, we repeated these experiments in the presence of other GABA_A_R and GABA_B_R antagonists: bicuculline methiodide (BMI, 20 μM) and phaclofen (PH, 20 μM), respectively. When BMI was washed onto PNs exhibiting HypOs, 3 out of 8 neurons transitioned to burst mode with cessation of HypOs. For non-bursting neurons, neither BMI, nor the consequent addition of PH, significantly impact HypO amplitudes (4-AP: −6.93 ± 2.98 mV, *n* = 5, 4-AP/BMI: −5.73 ± 1.48 mV, n = 5; paired *t*-test: *p* = 0.534; 4-AP/BMI/PH: −4.28 ± 3.87 mV, n = 5; paired t-test: *p* = 0.181) or frequency (4-AP: 0.08 ± 0.05 Hz, 4-AP/BMI: 0.04 ± 0.02 Hz; paired t-test *p* = 0.119; 4-AP/BMI/PH: 0.03 ± 0.03 Hz, n = 5; paired t-test: *p* = 0.169) (Fig. 2B–2Bii). Notably, in some cases at −55 mV, HypOs demonstrated a biphasic response with an initial fast depolarizing phase, followed by the slow hyperpolarizing phase. The depolarizing phase of these oscillations was reliably blocked by BMI (Fig. 2B middle trace, note disappearance of upward deflection over time). This suggests at −55 mV with a E_Cl_ = −49.48 mV, GABA_A_ currents have a depolarizing effect while the HypOs are not impacted by GABA_A_ receptor activation nor mediated by GABA_B_ receptor activation. Together, these data indicate that HypOs are not mediated by GABA receptor activation exclusively. Nevertheless, ictal-like activity (prolonged and repetitive bursting) can sometimes be triggered by a combination of 4-AP and GABA_A_/_B_ antagonization.

We next examined the role of glutamatergic transmission on these oscillations by determining the influence of AMPA and NMDA receptors. When we washed on a combination of DNQX (AMPAR antagonist, 20 μM) and D-APV (NMDAR antagonist, 50 μM) we found no significant changes in HypO amplitude (4-AP: −8.08 ± 2.58 mV, 4-AP/DNQX/DAPV: −7.47 ± 1.54 mV, n = 5; paired t-test: *p* = 0.581) (Fig. 2 Ci). Glutamatergic receptor blockade led to a nonsignificant reduction in HypO frequency that is similar to the natural decrease in HypO frequency (4-AP: 0.06 ± 0.04 Hz, 4-AP/DNQX/D-APV: 0.03 ± 0.01 Hz, n = 5; paired t-test, *p* = 0.131) (Fig. 2Cii). Thus, HypOs are network oscillations which are not solely mediated by GABAergic or glutamatergic transmission. We were surprised by these results, given that these events are synchronized between neighboring cells as shown by our paired recordings, and that GABAergic neurotransmission is often crucial for the generation of network oscillations. Additionally, we had hypothesized that PN firing, network excitability, and by extension, an increase in glutamatergic activity, would have contributed significantly to the generation of HypOs. However, the blockade of voltage-gated potassium channels by 4-AP may cause fluctuations in extracellular potassium, which could foster network activation regardless of glutamatergic neurotransmission.

### 3.4. Potassium currents mediate 4-AP-induced HypOs

**Fig. 3.**
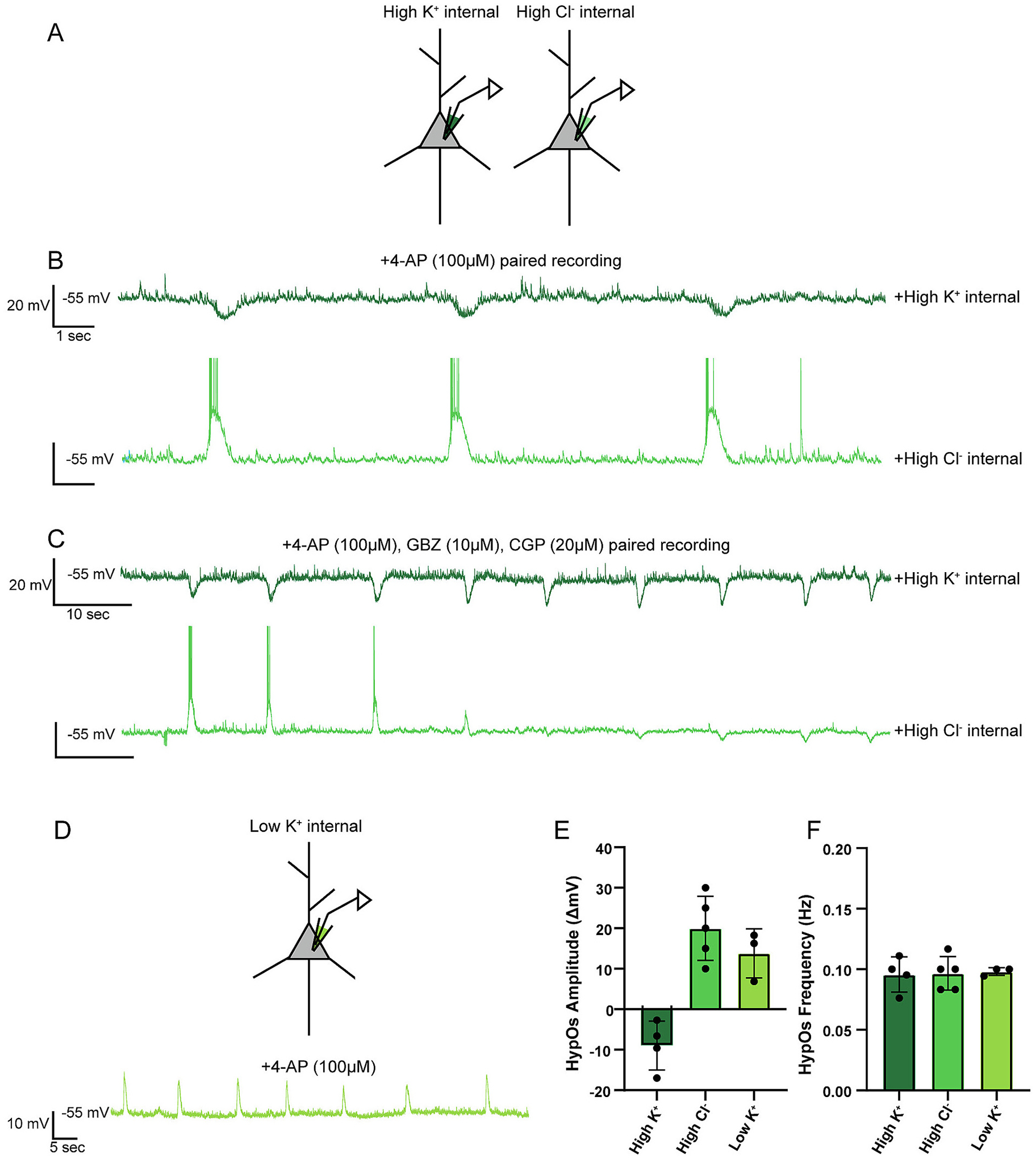
The reversal potential for HypOs is mediated by potassium. A) Schematic of paired whole-cell recordings from L2/3 PNs with high K^+^ internal in one neuron (dark green) and high Cl^−^ internal in one neuron (light green). B) Paired recording with 4-AP (100 μM) wash-on and different internal solutions (high K^+^ vs high Cl^−^) indicates that when chloride has a depolarized reversal potential, this ion contributes to bursting activity that is synchronized with HypOs. C) Paired recording demonstrates that the addition of GABA_A_ and GABA_B_ receptor blockers (GBZ, 10 μM and CGP, 20 μM, respectively) to a cell recorded with a high Cl^−^ internal causes bursting to terminate and HypOs to become unmasked. D, Top) Schematic of whole-cell recording with a Low K^+^ internal solution. D, Bottom) Example trace of L2/3 PN HypOs when a low K^+^ internal solution was used demonstrates that HypO is depolarizing when the K^+^ reversal potential is positive. E) HypO amplitudes (ΔmV) are dependent on the reversal potentials of both potassium and chloride (n_HighK+_ = 4, n_HighCl-_ = 5, n_LowK+_ = 3). F) HypO frequencies are not controlled by differences in chloride or potassium driving force (n_HighK+_ = 4, n_HighCl-_ = 5, n_LowK+_ = 3).

Our previous results suggest that both K^+^ and Cl^−^ may contribute to HypOs, given the reversal potential of these oscillations. Therefore, we examined the role of both ions by performing paired recordings with two separate internal solutions (*n* = 2 pairs, Fig. 3A). In one cell of the pair, we used our standard K-gluconate (high K^+^: E_K_ = −108.80 mV, E_Cl_ = −49.48 mV), and in the second cell of the pair, we used a KCl-based solution (high Cl^−^: E_K_ = −108.80 mV, E_Cl_ = +0.89 mV) to enhance the depolarizing potential of chloride. In the presence of 4-AP, the high K^+^ cell demonstrated typical HypO responses while the high chloride cell demonstrated bursting. These bursts were synchronized with the HypOs and had similar durations to the HypOs (approximately 1 s; Fig. 3B) suggesting that HypOs could be partially mediated by the influx of chloride ions. However, when we then added GBZ (10 μM) and CGP35348 (20 μM) to these recordings, the high Cl^−^ internal recording shifted from a bursting pattern to a typical HypO pattern (Fig. 3C, bottom trace) while the HypOs from the K-gluconate internal recording were unaffected (Fig. 3C, top trace). This suggests that GABA_A_R activation is triggered during HypOs, and that GABAergic interneuron synchrony plays a role in regulating these oscillations. However, neither GABA_A_-mediated chloride currents nor GABA_B_-mediated potassium currents were responsible for the remaining hyperpolarization.

We then investigated if a shift in potassium reversal potential could lead to a depolarization of the 4-AP-induced network oscillations. For these experiments, we used a cesium-based internal solution (Low K^+^, E_K_ = +84.90 mV, E_Cl_ = −49.48 mV). When we recorded using the low K^+^ internal solution, we found that all HypOs were depolarizing (13.800 ± 6.087 mV; *n* = 3; Fig. 3D–E). The different internal solutions did not affect the frequency of 4-AP-induced oscillations (Fig. 3F). Therefore, 4-AP-induced HypOs appear to rely mostly on efflux of potassium to exhibit the hyperpolarization that exists at more physiological concentrations (i.e., high K^+^ internal; Fig. 3E).

### 3.5. Inhibition of cation-chloride cotransporters results in a small reduction of HypO amplitudes

Based on these findings, we postulated that cation-chloride cotransporters (CCCs) might play a role in generating these oscillations. In neurons, two CCCs act in concert to establish the intracellular concentrations of potassium and chloride: the Na^+^-K^+^−2Cl^−^ cotransporter, NKCC1, and the neuron-specific K^+^-Cl^−^ cotransporter, KCC2 (Huang et al., 2012; Loscher et al., 2013; Shimizu-Okabe et al., 2011; Talos et al., 2012; Tyzio et al., 2009; Wu et al., 2019). In neonatal mammals, elevated levels of NKCC1 and low levels of KCC2 lead to high [Cl^−^]_i_ and a depolarizing effect of GABA (Ben-Ari et al., 1989). Once mature, neurons express lower levels of NKCC1 and higher levels of KCC2, leading to low [Cl^−^]_i_ and hyperpolarizing GABA (Ben-Ari and Cherubini, 2022; Dzhala et al., 2005; Rivera et al., 1999). Previous studies have demonstrated that epilepsy and other neurological disorders may lead to a developmental regression in NKCC1/KCC2 expression, resulting in excitatory GABA.

**Fig. 4.**
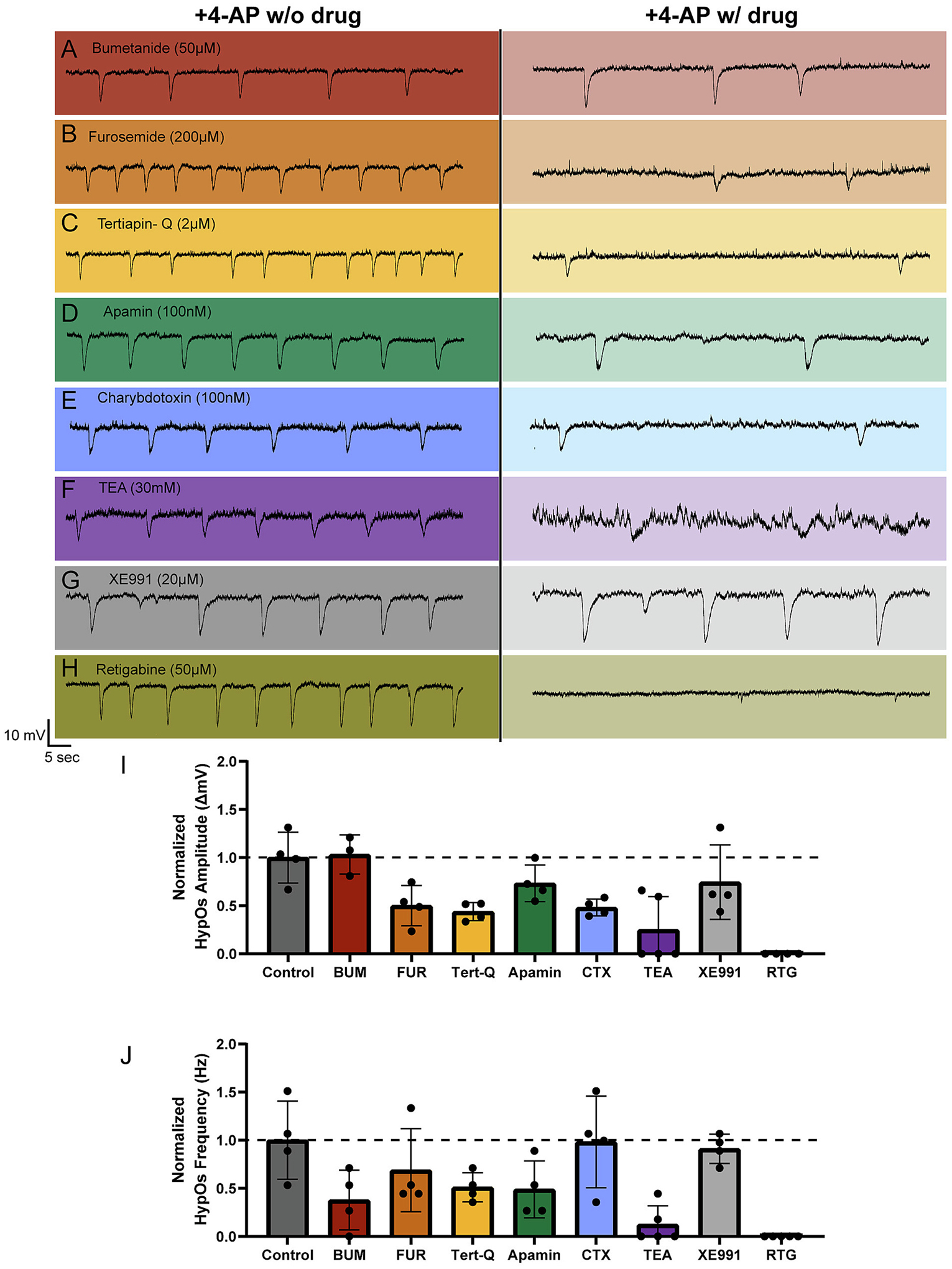
Potassium channel modulators perturb HypOs to varying degrees. A-H) Example traces of 4-AP induced HypOs before (left) and after (right) K^+^ channel modulator wash-on. I) Normalized HypO amplitudes before and after K+ channel blocker wash-on indicates retigabine (50 μM, n = 5) and to varying degrees TEA (30 mM, n = 3/5) are the only K^+^ channel blockers capable of eliminating HypOs. J) Normalized HypO frequencies before and after K^+^ channel blocker wash-on indicates reduction in HypO frequency due to K^+^ channel blockers, substantial frequency reduction with TEA (n = 5), and elimination of HypOs with retigabine (n = 5). Acronyms: Bumetanide = BUM, Furosemide = FUR, Tertiapin-Q = Tert-Q, Charybdotoxin = CTX, Tetraethylammonium = TEA, Retigabine = RTG.

We hypothesized that NKCC1 channels might contribute to the generation of HypOs. However, when we examined the effect of the NKCC1 channel blocker, bumetanide (BUM, 10–50 μM), we found only minimal changes when compared to the changes seen over time when slices are incubated in 4-AP alone (BUM (50 μM), n = 3: 15.4 % increase in amplitude, 67.9 % decrease in frequency; CON, *n* = 4: 25.8 % increase in amplitude, 52.1 % decrease in frequency; Fig. 4A, I–J, Supplementary Fig. 3 A). We next looked at blockade of KCC2 with furosemide (FUR, 200 μM), as it has demonstrated some anti-ictal effects in vitro (Loscher et al., 2013; Viitanen et al., 2010). FUR showed meaningful reduction of HypO amplitude compared to control but negligible effect on HypO frequency (61.3 % reduction, *n* = 4; Fig. 4B, I–J, Supplementary Fig. 3B). Therefore, we postulate that blockade of KCC2 channels may decrease [K^+^]_o_, resulting in smaller HypO amplitudes; however, furosemide has many off target effects including NKCC1 channel blockade (Chen et al., 2022). Nevertheless, 4-AP-induced HypOs are not generated through acute blockade of CCCs.

### 3.6. Blockade of potassium channels reduces but does not eliminate HypOs

Given that slight changes in [K^+^]_o_ can greatly influence neuronal excitability, it is not surprising that perturbations in a variety of potassium channels have been implicated in the generation of seizures. Potassium channel mutations have been demonstrated in a multitude of human epilepsy syndromes and are causative of epilepsy in animal models (Kohling and Wolfart, 2016; Villa and Combi, 2016). Thus, we systematically assessed the contribution of different classes of potassium channels to the generation of HypOs in human tissue. GIRK channels are activated by G-protein-linked neurotransmitter receptors including GABA_B_ receptors, dopamine (D2) receptors, adenosine A1 receptors, and others. Given that the HypOs resemble GABA_B_-mediated currents, but are not eliminated by blockade of GABA_B_ receptors, we hypothesized that GIRK channels might underlie the HypOs. To do this, we blocked Kir channels, including GIRK channels, with Tertiapin-Q (Tert-Q, 2 μM). Tert-Q reduced HypO amplitude by 30.7 % and frequency by 62.2 % (n = 4), indicating it had a reduced effect compared to furosemide (Fig. 4C, I–J, Supplementary Fig. 3C).

Both classes of calcium-activated potassium channels, the small conductance (SK) and the large-conductance (BK) channels can contribute to overall network excitability via their roles in generating action potential afterhyperpolarizations (AHPs) (Avoli and Olivier, 1989; Faber and Sah, 2002; Higgs and Spain, 2009; Lorenzon and Foehring, 1992; Zang et al., 2018). We found that blocking SK channels with apamin (100 nM) had a minimal influence on HypO amplitude (9.8 % reduction) and reduced the frequency by 70.2 % (*n* = 4). Compared to the control frequency reduction of 52.1 %, this finding suggests that SK channels do contribute to HypO generation (Fig. 4D, I–J, Supplementary Fig. 3D) (Blatz and Magleby, 1986). Interestingly, when a blocker of BK channels, TEA (30 mM), was used we observed a complete elimination of HypOs in 3/5 PNs, and 1/5 PNs shifted from HypOs to repetitive bursting activity (*n* = 5; Fig. 4F, I–J, Supplementary Fig. 3F). However, it is essential to note that TEA is a non-specific potassium channel blocker and can affect multiple classes of potassium channels in addition to BK channels (Mathie et al., 1998; Soh et al., 2022; Wulff and Zhorov, 2008). Therefore, we also assessed a more specific BK channel blocker, charybdotoxin (CTX). With CTX (100 nM), we observed a 39.8 % reduction in HypO amplitude, like furosemide (n = 4). However, CTX (100 nM) only reduced HypO frequency by 36.6 %, which is even less than what was observed after 9–12 min of 4-AP wash-on alone control and less than what was observed using apamin (Fig. 4E, I–J, Supplementary Fig. 3E). Together, these data indicate that HypOs are dependent on potassium channels, but are not primarily generated by GIRK channels, SK channels, or BK channels.

### 3.7. Kv7.2- Kv7.5 (KCNQ2–5) channel activation eliminates 4-AP-induced HypOs

Our results demonstrate that TEA had robust effects on HypO frequency and amplitude. KCNQ channels represent an important class of potassium channels that are blocked by TEA (Hadley et al., 2000). KCNQ2 and KCNQ3 channels are known to mediate the M-type potassium current, which is a slow, non-inactivating potassium current (Brown and Adams, 1980; Brown and Passmore, 2009). Activation of the M-current can stabilize the membrane potential, inhibit the initiation of action potentials, and decrease the probability of burst firing (Borgini et al., 2021; Carver and Shapiro, 2019; Knight et al., 2023; Kobayashi et al., 2008; Peretz et al., 2005; Schroeder et al., 2000; Yang et al., 2022). Interestingly, KCNQ2 and KCNQ3 mutations have been found in human epilepsies including developmental and epileptic encephalopathy (DEE) and benign familial neonatal epilepsy (BFNE) (Arredondo et al., 2022; Cooper et al., 2000; Dedek et al., 2001; Edmond et al., 2022; Kim et al., 2021a; Kim et al., 2021b; Knight et al., 2023; Malerba et al., 2020; Nardello et al., 2020; Olson et al., 2017; Schroeder et al., 1998; Shellhaas et al., 2017; Singh et al., 1998).

Given the slow kinetics of KCNQ channels and the association with hyperexcitability, we assessed the effect of M-current modulation on HypOs. With the addition of the KCNQ channel blocker XE991 (20 μM), we observed only a 20.5 % reduction in HypO amplitude (n = 4). Additionally, XE991 led to only a small reduction in HypO frequency (43.7 %) that was similar to the reduction with time in 4-AP only (Fig. 4G, I–J, Supplementary Fig. 3G). This could mean that HypOs do not rely upon KCNQ channels for potassium efflux. Alternatively, the robust effects of TEA could arise from nonspecific effects on a variety of potassium channels. However, given the clear association of potassium channels with the HypOs, we decided to evaluate whether the activation of KCNQ channels with retigabine (RTG) would amplify HypOs. Interestingly, the activation of KCNQ2–5 channels with RTG (50 μM) led to a complete elimination of HypOs (paired *t*-test: amplitude: *p* = 0.001; frequency: *p* = 0.0003, *n* = 5; Fig. 4H, I–J, Supplementary Fig. 3H). The only other drug that abolished these oscillations so far was TTX (1 μM) (Fig. 2). We suggest that RTG could be working similarly by reducing the ability for neurons to burst or repetitively fire, which would stabilize [K^+^]_o_. Another possibility is that KCNQ2–5 channel activation may counteract the influence of blocking voltage-gated potassium channels caused by 4-AP by expelling [K^+^]_i_ slowly.

### 3.8. Intracellular calcium influences HypOs

**Fig. 5.**
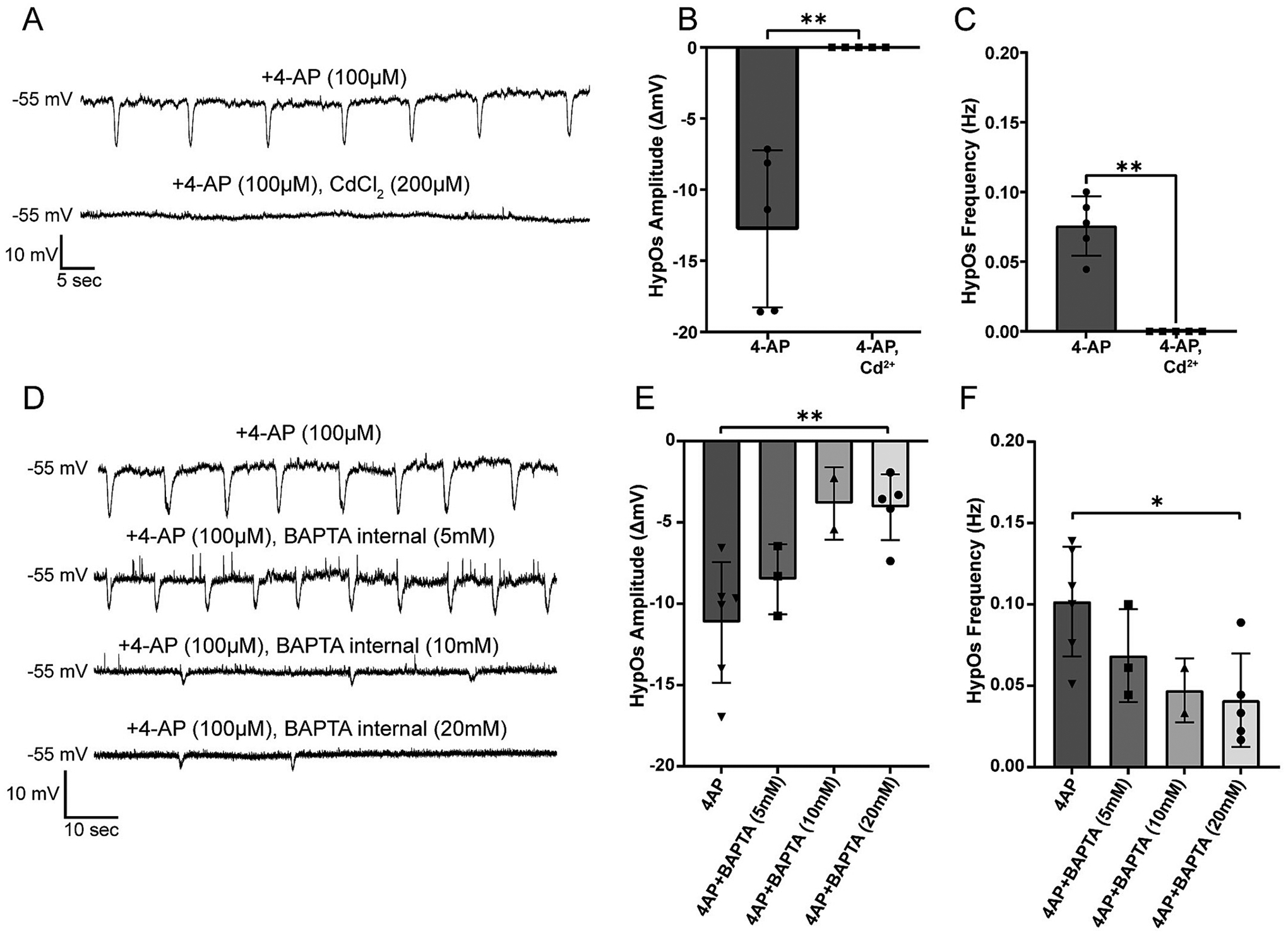
Voltage-gated Ca^2+^ channel blockage and chelation of internal Ca^2+^ diminish HypOs. A) Example traces of HypOs in the presence of 4-AP (100 μM) and 4-AP with CdCl_2_ (200 μM) demonstrates that Cd^2+^ can eliminate HypOs by blocking voltage-gated calcium channels. B, C) HypOs are completely abolished and thus both amplitude and frequency are reduced to zero in the presence of CdCl_2_ (paired t-test: amplitude: *p* = 0.007, frequency: *p* = 0.0014, n = 5). D) Example traces of 4-AP + internal BAPTA salt internal (5 mM, 10 mM or 20 mM) influence on HypOs. *E*-F) Calcium chelation with 20 mM of intracellular BAPTA leads to a significant reduction in HypO amplitude and frequency (MWU test: amplitude: *p* = 0.009, frequency: *p* = 0.017, n = 5). Lower concentrations of intracellular BAPTA lead to smaller effects.

Transient changes in intracellular calcium can regulate neuronal excitability by affecting neuronal firing patterns, altering the probability of neurotransmitter release, and activating second messenger systems (Steinlein, 2014; Xu and Tang, 2018). Previous reports have demonstrated that 4-AP-induced oscillations can be eliminated with cadmium (Cd^2+^), a nonselective calcium channel blocker (Levinson et al., 2020). We found that CdCl_2_ (200 μM) completely abolished HypOs in epileptic human tissue (Fig. 5A–C, amplitude 4-AP: −12.75 ± 5.52 mV, 4-AP/CdCl_2_: 0 mV, n = 5; paired t-test: *p* = 0.007; frequency 4-AP: 0.07 ± 0.02 Hz, 4-AP/CdCl_2_: 0 Hz, n = 5; paired t-test: *p* = 0.0014). To differentiate between pre- and post-synaptic influences of calcium on HypOs, we added intracellular BAPTA at various concentrations to chelate intracellular calcium (Huang et al., 2012). We observed a significant reduction in the HypO amplitude and frequency with 20 mM of intracellular BAPTA compared to the HypOs recorded from the same tissue without any intracellular BAPTA (Fig. 5D–F amplitude 4-AP: −11.14 ± 3.71 mV, *n* = 6; 4-AP/BAPTA_20mM_: −4.07 ± 2.02 mV, n = 5; MWU: *p* = 0.009; frequency 4-AP: 0.10 ± 0.03 Hz, n = 6; 4-AP/BAPTA_20mM_: 0.04 ± 0.03 Hz, n = 5; MWU: *p* = 0.017). Lower concentrations of BAPTA also resulted in lesser reductions in amplitude and frequency of the HypOs (Fig. 5D–F). Thus, postsynaptic intracellular calcium dynamics play a significant role in 4-AP-induced HypOs.

### 3.9. Gap junctions facilitate synchronization to regulate 4-AP induced HypOs

We have presented evidence demonstrating that HypOs are synchronous network events that are not mediated by glutamatergic or GABAergic neurotransmission and are modulated by potassium channels and intracellular calcium. Gap junctions are known to regulate electrical synchronization of GABAergic interneurons and play a role in buffering extracellular ion concentrations (Bedner et al., 2015; Deshpande et al., 2017; Draguhn et al., 1998; Hu and Agmon, 2015; Jin and Chen, 2011; Johnson et al., 2017; Via et al., 2022; Wu et al., 2018; Yu and Santhakumar, 2023). Thus, we hypothesized that gap junctions could play a role in synchronizing network oscillations. Unfortunately, most pharmacologic modulators of gap junctions have off-target effects (Manjarrez-Marmolejo and Franco-Pérez, 2016). Therefore, we assessed the effects of multiple gap junction blockers, including a non-specific gap junction blocker, carbenoxolone (CBX) and three putative neuronal gap junction blockers, niflumic acid (NFA) and meclofenamic acid (MFA), and mefloquine (MFLQ) (Manjarrez-Marmolejo and Franco-Pérez, 2016; Rossokhin et al., 2019; Volnova et al., 2022).

**Fig. 6.**
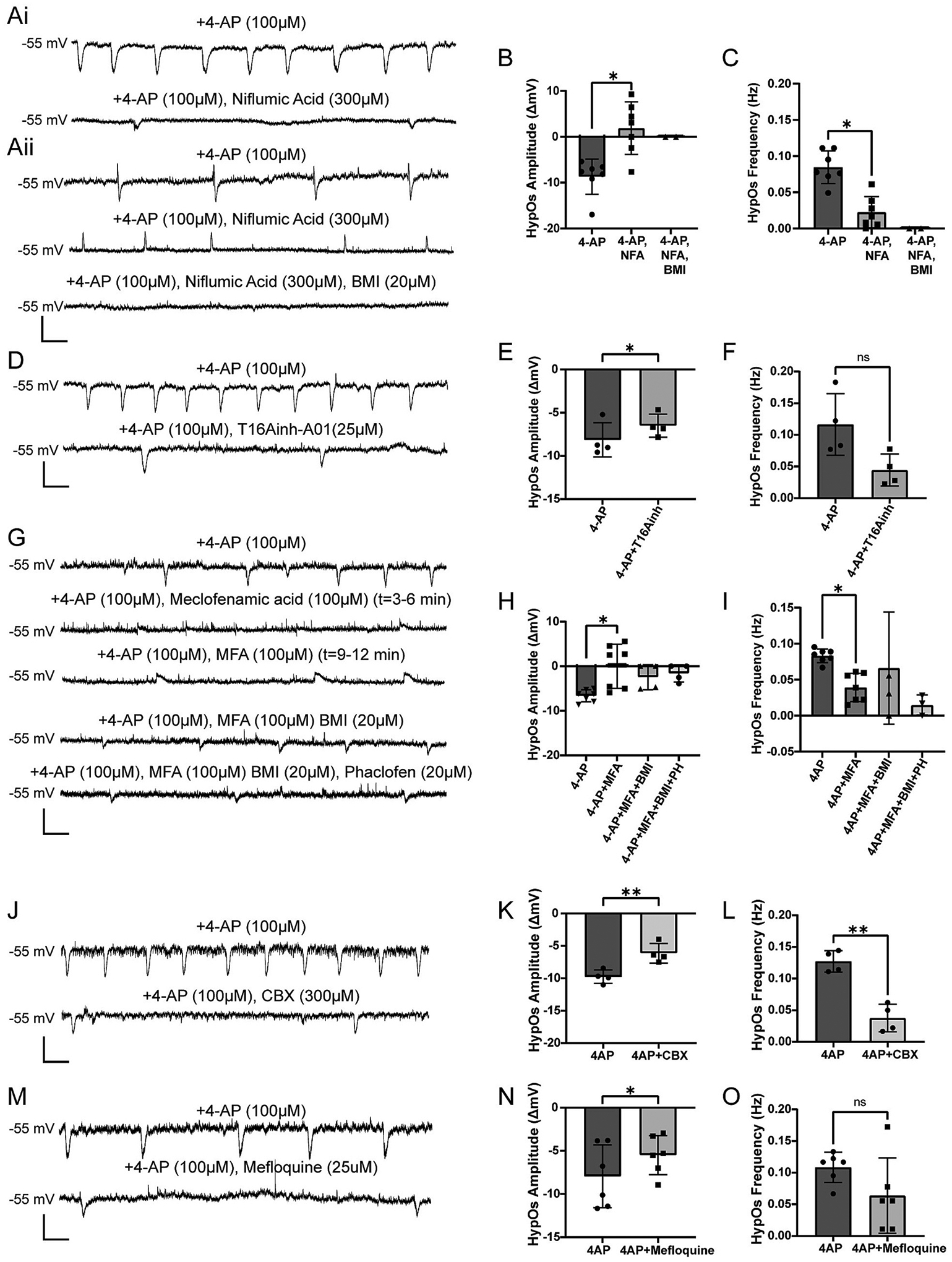
Gap junctions influence HypOs. Ai) Example traces of 4-AP (100 μM) with NFA (300 μM) wash-on causing a reduction in HypO amplitude. Aii) Example traces showing NFA leading to a reversal of polarity in 4-AP induced HypOs, and the termination of HypOs with consecutive addition of BMI (20 μM). B, C) NFA caused a significant reduction in HypO amplitude and frequency (Wilcoxon test: amplitude: *p* = 0.016, frequency: *p* = 0.016, *n* = 7). D) Example traces of the effects of T16Ainh-A01 (25 μM) on HypOs. E, F) T16Ainh-A01 caused a significant reduction in HypO amplitude but did not significantly affect frequency (paired t-test: amplitude: *p* = 0.048, frequency: *p* = 0.083, *n* = 4). G) Example traces of 4-AP showing the effects of MFA (100 μM) alone or in combination with bicuculline methiodide (BMI, 20 μM), and Phaclofen (PH, 20 μM). Note that the changes in MFA are progressive over time. H, I) MFA (100 μM) demonstrates similar effects to NFA and significantly decreases HypO amplitude and frequency (Wilcoxon test: amplitude: *p* = 0.016, frequency: *p* = 0.016, n = 7). Additionally, MFA can cause polarity switch to depolarizing potentials (n = 4/7). J) Example traces of the influence of carbenoxolone (CBX, 300 μM) on HypOs. K, L) CBX causes a significant reduction in HypO amplitude and frequency (paired t-test: amplitude: *p* = 0.006, frequency: *p* = 0. 006, n = 4). M) Example traces of the influence of mefloquine (25 μM) on HypOs. N,O) Mefloquine causes a significant decrease in HypO amplitude but does not affect frequency (paired t-test: amplitude: *p* = 0.022, frequency: *p* = 0.302, n = 4). Scale bars: 10 mV, 5 s.

The addition of NFA (300 μM) led to elimination of HypOs in 1/7 PN, reduction of HypO amplitude in 2/7 PNs, and reversal of HypO polarity in 4/7 PNs (Fig. 6A–C, 4-AP: −8.69 ± 3.83 mV; 4-AP/NFA: 1.88 ± 5.72 mV, *n* = 7; Wilcoxon test: *p* = 0.016). NFA also significantly reduced HypO frequency in these cells (Fig. 6C, 4-AP: 0.08 ± 0.02 Hz; 4-AP/NFA: 0.02 ± 0.02 Hz, n = 7; Wilcoxon test: *p* = 0.016). To rule out any off-target effect of NFA on calcium-activated chloride channels (Huang et al., 2012), we tested the effect of the specific CACC blocker, T16-Ainh-A01 (25 μM). T16-Ainh-A01 significantly influenced HypO amplitude but did not influence frequency (Fig. 6E–F, amplitude 4-AP: −8.13 ± 1.98 mV, 4-AP/T16: −6.50 ± 1.33 mV, *n* = 4; paired *t*-test: *p* = 0.048; frequency 4-AP: 0.12 ± 0.05 Hz, 4-AP/T16: 0.04 ± 0.02 Hz, n = 4; paired t-test: *p* = 0.083) (Fig. 6E–F). This indicated that the polarity switch caused by NFA was not through CACCs.

The addition of MFA (100 μM) to the perfusate led to similar results as the addition of NFA: some cells exhibited a polarity switch of the HypO and most cells demonstrated a significant reduction in HypO frequency (Fig. 6G–I, amplitude 4-AP: −6.62 ± 1.35 mV, 4-AP/MFA: 0.03 ± 4.94 mV, n = 7; Wilcoxon test: *p* = 0.016; frequency 4-AP: 0.08 ± 0.01 Hz, 4-AP/MFA: 0.04 ± 0.02 Hz, n = 7; Wilcoxon test: *p* = 0.016). In the L2/3 PNs where NFA or MFA led to depolarizing oscillations, we washed on BMI (20 μM), which completely eliminated the depolarizing oscillations. The addition of BMI with MFA led to a return of hyperpolarizing HypOs in 2/4 cells and caused a transition to spontaneous firing in the remaining 2/4 cells (Fig. 6A–C, 6G–I). In contrast, the addition of PH (20 μM) did not eliminate the HypOs that were unmasked after MFA and BMI wash-on (Fig. 6G–I). These results echo what we demonstrated in Fig. 2B: when HypOs exhibit leading depolarization, blockade of GABA_A_Rs can eliminate the depolarizing phase. Therefore, we believe this HypO polarity switch is due to effects of NFA and MFA that are separate from their effects of GABA_A_Rs.

NFA and MFA both have significant effects on KCNQ channels in addition to their known effects on gap junctions. To separate the effects of KCNQ channel activation from gap junction blockade, we examined the influence of CBX, which is not known to affect KCNQ channel activity. We found that the addition of CBX (300 μM) significantly reduced HypO amplitude and frequency without completely eliminating these oscillations (Fig. 6J–L, amplitude 4-AP: −9.75 ± 1.05 mV, 4-AP/CBX: −6.13 ± 1.51 mV, n = 4; paired t-test: *p* = 0.006; frequency 4-AP: 0.13 ± 0.02 Hz, 4-AP/CBX: 0.04 ± 0.02 Hz, n = 4; paired t-test: *p* = 0. 006). To further elucidate if gap junction blockade was the main effect of these notoriously nonspecific gap junction modulators, we also evaluated the effects of MFLQ (25 μM) on HypOs. MFLQ has no reported history of impacting KCNQ2–5 channels and has selective affinity for connexins expressed by cortical interneurons (Connors, 2012; Jin and Chen, 2011; Lee et al., 2021; Manjarrez-Marmolejo and Franco-Pérez, 2016). We found that MFLQ caused a significant decrease in HypO amplitude but no significant change in frequency (Fig. 6M–O, amplitude 4-AP: −10.00 ± 2.25 mV, 4-AP/MFLQ −6.62 ± 1.56 mV, n = 4; paired t-test: *p* = 0.022; frequency 4-AP: 0.10 ± 0.02 Hz, 4-AP/MFLQ: 0.06 ± 0.08 Hz, n = 4; paired t-test: *p* = 0.302). Together, these data suggest that gap junctions, most likely electrically coupling cortical interneurons or astrocytes, partially regulate 4-AP-induced interictal-like activity possibly by decreasing prolonged neuronal bursting and desynchronizing GABAergic interneurons or decreasing epileptiform activity in astrocytes, respectively (Gigout et al., 2006; Kohling et al., 2001; Onodera et al., 2021; Volnova et al., 2022).

## 4. Discussion

**Fig. 7.**
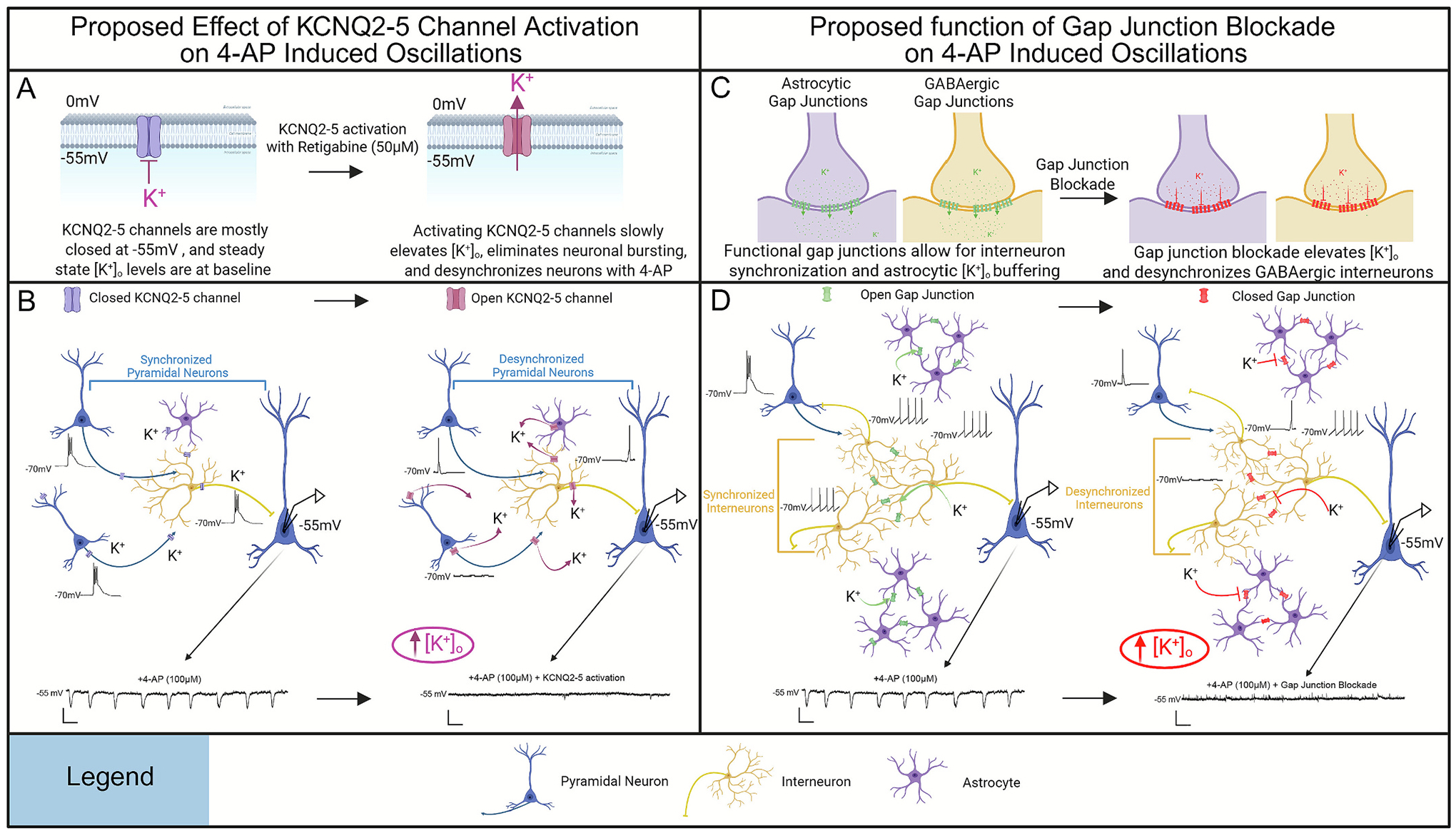
Schematic of the proposed mechanism of how KCNQ2–5 channel activation and gap junction blockade mediate 4-AP induced HypOs. A-B) KCNQ channels are not open at −55 mV and would not suppress 4-AP induced neuronal bursting or synchronization. Activation of KCNQ2–5 channels allows for slow, m-current to attenuate neuronal bursting, network synchronization, and 4-AP induced HypOS. C–D) Functional gap junctions in astrocytes allow effective buffering of [K+]_o_ and allow interneurons to synchronize electrically. With blockade of gap junctions, inhibitory interneurons are less likely to form synchronous network activity. Moreover, gap junction blockade inhibits astrocytic [K+]_o_ buffering leading to an overall increase in [K+]_o_. Overall, this would lead to reduction in 4-AP induced HypOs.

In the present study, we utilized cortical tissue resected from pediatric patients with epilepsy or deep-seated tumors to investigate the effects of the proconvulsant drug, 4-AP, on L2/3 PNs. We have demonstrated that 4-AP reliably induces robust slow hyperpolarizing oscillations, which we term HypOs, in L2/3 PNs. These oscillations are synchronized between neurons, are not generated by glutamatergic or GABAergic neurotransmission alone, require network activity, and require intracellular potassium and calcium. Additionally, we show that epileptic human and mouse neurons exhibit larger HypOs than control neurons. We also show that 4-AP-induced HypOs are mediated by network driven increases in [K^+^]_o_. These oscillations can be eliminated with activation of KCNQ2–5 (K_v_7.2-K_v_7.5) channels (Fig. 7A, B). Finally, these potassium fluctuations are partially mediated by gap junction synchronization (Fig. 7C, D).

When exploring the mechanisms underlying the generation of HypOs, our first hypothesis was that GABAergic currents were the source of these oscillations. Not only are many physiological oscillations (such as gamma oscillations) mediated by GABAergic activity, but prior studies have shown that pathological oscillations similar to HypOs are heavily dependent on GABA. For example, one prior study showed that human cortical oscillations, induced by 4-AP, were reduced by GABA_A_R blockade and transformed into paroxysmal bursting discharges in the presence of GABA_A_/GABA_B_R blockade (Levinson et al., 2020). That study suggested that GABA_B_ receptors play a significant role in the interictal to ictal transition. We similarly found that GABA receptor blockade contributes to PN bursting. However, GABA_A_R or GABA_B_R blockade did not eliminate HypOs despite trying multiple antagonists at varying concentrations. Thus, GABAergic neurotransmission occurs during these network level events but does not dictate the hyperpolarizing oscillation. Instead, GABAergic neurotransmission possibly gates the interictal to ictal transition by regulating synchrony between glutamatergic neurons and shunting additive excitatory inputs. Moreover, we propose that GABAergic interneurons regulate HypOs through gap junctions, synchronized firing, and synchronized potassium fluctuations. We suggest that future studies should be done to directly investigate the role that interneurons play in generating HypOs.

We next turned our attention to cation-chloride cotransporters (CCCs), as previous research has shown that blockade of NKCC1 channels with bumetanide (8 μM) suppressed interictal discharges (IIDs) in 9 of 12 FCD slices, and we found that the 4-AP induced oscillations were between chloride and potassium reversal potentials (Blauwblomme et al., 2019). We found that at the lowest dose tested, 10 μM, when bumetanide would be most specific to NKCC1 channels (Payne et al., 2003), bumetanide was unable to reduce HypO amplitudes after 20 min of wash-on (*n* = 1) in FCD Type IIA tissue (age = 5 y.o.). We did not test this concentration consecutively across slices due to our desire to find a treatment that eliminated HypOs robustly across epilepsy etiologies. Furthermore, at this concentration of bumetanide, it is postulated that extracellular levels of potassium would be elevated leading to a reduction in 4-AP HypO amplitudes, however, NKCC1 channel blockade did not have this effect. This possibly could be due to age, as bumetanide is thought to influence seizure activity neonatally, or due to the influence of NKCC1 channel blockade being specific to epilepsy subtypes (Kahle and Staley, 2008; Talos et al., 2012; Tyzio et al., 2009). Further increases in bumetanide concentration (20–50uM) also did not eliminate HypOs, but this could be due to overlap with KCC2 blockade. Furosemide (200 μM) was used as a blocker of KCC2 channels, although it also is able to block NKCC1 channels (Loscher et al., 2013). We found marked reduction of HypO amplitudes (61.3 % reduction) with acute application of furosemide (200 μM) suggesting while NKCC1 channels and KCC2 channels are not responsible for 4-AP induced HypOs, alterations in cortical potassium/chloride homeostasis underlie network driven events (Gonzalez et al., 2018). Future studies should be done to investigate whether longer-term modulation of NKCC1 and KCC2 channels influence ictal or interictal-like activity induced by 4-AP. More robust modulation of the potassium/chloride cotransporters may be required to alter the homeostasis of these ions enough to modulate HypOs.

We next demonstrated that a hyperpolarizing driving force of potassium is required for HypOs to be hyperpolarizing and that potassium is a key modulator of these currents (Fig. 3). This was backed up by a non-specific potassium channel blocker, TEA, leading to significant reduction of HypO amplitude and frequency. In terms of finding a complete mitigator of the 4-AP induced fluctuations in extracellular potassium, we showed that activation, but not blockade, of KCNQ2–5 (Kv7.2- Kv7.5) channels with retigabine completely eliminated HypOs. Although this may at first seem counter-intuitive, we propose that XE991, the KCNQ channel blocker, may not be able to completely block KCNQ channels at the membrane potential we used for recording, as previously reported (Greene et al., 2017). Alternatively, KCNQ2–5 channels may not be major driver of HypO K^+^ potentials. Instead, the KCNQ2–5 channel activator, retigabine, may cause HypO blockade via its known mechanism of allowing KCNQ opening at physiological membrane potentials (Wuttke et al., 2005). Open KCNQ channels will lead to an overall decrease in neuronal firing, synaptic activity, and bursting, in turn leading to the cessation of network-driven HypOs.

Retigabine, also known as ezogabine, was initially developed as an anti-seizure medication, and was approved in both the US and Europe in 2011 (Brickel et al., 2020; Dailey et al., 1995). Retigabine showed good efficacy for patients with epilepsy but was taken off the market in 2017 due to significant side effects, notably the fingernails and sclera turning blue (Brickel et al., 2020; Kuersten et al., 2020). KCNQ2 mutations can be causative of epilepsy, including in patients with benign familial neonatal convulsions (Singh et al., 1998). In patients with KCNQ2 mutations, retigabine is an especially effective treatment for seizures and also leads to neurodevelopmental improvement (Knight et al., 2023; Nissenkorn et al., 2021). Moreover, previous work has indicated that the addition of retigabine leads to cessation of neuronal bursting, 4-AP induced bursts, spontaneous sharp waves, and epileptiform activity (Armand et al., 2000; Straub et al., 2001; Yaari et al., 2007; Yonekawa et al., 1995). At least two other KCNQ-activating drugs: NS15370 and ICA-27243 have demonstrated potent anticonvulsant effects in rodent models of epilepsy (Dalby-Brown et al., 2013; Large et al., 2012; Roeloffs et al., 2008), we feel that the investigation of other KCNQ channel activator that other KCNQ activators should be investigated therapeutically given the robust effect that KCNQ activation has on the hyperexcitability of ex vivo human tissue. Indeed, there is a current phase 3 trial of BHV-7000, a KCNQ2 channel activator, for adults with idiopathic generalized epilepsy. (https://clinicaltrials.gov/study/NCT06425159).

We also found that intracellular calcium fluctuations regulate HypOs, as calcium chelation significantly reduced HypO amplitude. Calmodulin is a major regulator of calcium signaling in neurons and interestingly this protein is an essential auxiliary subunit of KCNQ2/3 channels (Wen and Levitan, 2002). Calmodulin inhibits activation of KCNQ2–5 channels when intracellular calcium levels increase (Chang et al., 2018; Zhuang and Yan, 2020). We thereby postulate that internal calcium chelation inactivates calmodulin, which would then allow KCNQ2–5 channels to be active, ultimately reducing [K^+^]_o_ fluctuations.

The fact that HypOs were differentially regulated by various gap junction blockers is most likely due to their off-target effects. Additionally, these drugs have varying specificity for neuronal versus astrocytic connexin hemichannels (Manjarrez-Marmolejo and Franco-Pérez, 2016). For example, NFA blocks CACCs, potentiates GABA, shifts the activation curve of KCNQ5 and inhibits Kv1.1 channels (Lee and Wang, 1999; Rossokhin et al., 2019; Schroeder et al., 2000; Sharonova and Dvorzhak, 2013; Zhang et al., 2015). MFA similarly potentiates GABA, shifts the activation curve of KCNQ2/3 channels, and inhibits Kv2.1 channels (Barber et al., 2017; Franco-Pérez, 2016; Jin and Chen, 2011; Peretz et al., 2005). CBX and MFLQ are not known to impact KCNQ channels but do have other off-target effects (Bazzigaluppi et al., 2017; Gigout et al., 2006; Jin and Chen, 2011; Kohling et al., 2001; Nilsen et al., 2006; Volnova et al., 2022). Therefore, it is difficult to distinguish exactly how these gap junction blockers work to reduce 4-AP-induced HypOs.

One possibility is that neuronal gap junction blockade leads to a disconnection of GABAergic networks, reducing network oscillatory activity. Alternatively, the uncoupling of astrocytic gap junctions could lead to reduced ability of astrocytes to buffer [K^+^]_o_, resulting in an overall increase in [K^+^]_o_. Moreover, previous work has found that connexin 36, which is highly expressed in GABAergic interneurons, is not critical for the generation of epileptiform discharges in GABAergic networks and that the antiepileptic effects of CBX are likely to be due to blockade of GABA_A_ receptors (Beaumont and Maccaferri, 2011; Connors, 2012). Therefore, we suggest the differences in 4-AP HypO polarity switches between NFA, MFA, CBX, and MFLQ are most likely due to overlap with KCNQ2–5 channel activation and GABAergic neurotransmission versus gap junction blockade.

## 5. Conclusions

Given these results, we propose the following mechanism for the generation of HypOs in the presence of 4-AP: 4-AP leads to increased network activity via increased neuronal excitability and neurotransmitter release, which in turn, generates synchronous activity in GABAergic interneurons. The synchronization of GABAergic interneurons is generated via both GABAergic neurotransmission and gap junctions. The overall increased network activity additionally leads to local extracellular potassium fluctuations, which directly cause the HypOs by altering the resting membrane potential of glutamatergic neurons. With increased activation of KCNQ2–5 channels during this oscillatory activity, neuronal bursting and repetitive firing are reduced, abolishing the K^+^ fluctuations and preventing the generation of HypOs. Therefore, 4-AP-induced oscillations are due to GABAergic interneuron synchronizations leading to local microcircuit K^+^ fluctuations without the need for GABA neurotransmission.

## Supplementary Material

Supplementary Data

## Figures and Tables

**Table 1:** Patient Information. Pathological diagnosis, resected brain area, patient age and sex.

Pathological Diagnosis	Resection Location	Patient Age	Patient Sex
Patients with Drug-Resistant Epilepsy			
Chaslin’s subpial gliosis	Temporal lobe	4	F
Chaslin’s subpial gliosis	Temporal lobe	16	M
Chaslin’s subpial gliosis	Parietal Lobe	11	M
Gliosis	Temporal lobe	3	M
Gliosis	Temporal lobe	5	M
Low-grade glial neoplasm	Temporal lobe	1	F
Low-grade glioma: WHO grade 1	Temporal lobe	8	M
DNET: WHO grade 1	Frontal lobe	6	F
FCD type IIa	Frontal lobe	2	F
FCD type IIa	Frontal lobe	4	M
Tuberous sclerosis	Temporal, parietal lobe	2	M
Chaslin’s subpial gliosis	Temporal lobe	15	M
Gliosis	Temporal lobe	20	M
Gliosis	Temporal lobe	12	M
Ganglioglioma: WHO grade 1	Temporal lobe	13	F
Rasmussen Encephalitis	Temporal, frontal lobe	4	M
Hippocampal sclerosis	Temporal lobe	3	M
Ganglioglioma, WHO grade 1	Temporal lobe	3	M
FCD type IIa	Parietal lobe	5	F
Chaslin’s subpial gliosis	Temporal lobe	12	F
Diffuse low-grade glioma	Temporal lobe	1	F
Mild subpial gliosis	Temporal lobe	4	F
Mild Chaslin’s subpial gliosis	Occipital lobe	17	F
FCDIIID	Frontal, parietal lobe	12	M
Tuberous Sclerosis Complex	Frontal Lobe	5	M
FCD type 1	Temporal lobe	7	F
FCD type IIIB & DNET: WHO grade 1	Parietal lobe	10	F
FCD type 1A	Temporal Lobe	21	F
Tuberous sclerosis	Occipital Lobe	6	M
FCD type IIIc & Sturge-Weber Syndrome	Temporal Lobe	1	M
Gliosis	Temporal Lobe	16	F
Gliosis	Parietal Lobe	4	F
MOGHE	Frontal Lobe	8	M
Mild MCD	Temporal Lobe	9	M
Gliosis	Temporal Lobe	19	M
Low-grade glioneuronal tumor	Temporal Lobe	6	F
Ganglioglioma: WHO grade 1	Temporal Lobe	17	F
DNET: WHO grade 1	Temporal Lobe	16	M
Tuberous Sclerosis	Frontal Lobe	4	F
Control Patients			
Germinoma	Parietal Lobe	11	M
High-grade neuroepithelial tumor consistent with CNS embryonal tumor	Temporal Lobe	9	M
Vascular lesion, consistent with capillary-venous malformation and organizing hemorrhage	Temporal Lobe	12	F
Infant-type hemispheric glioma	Temporal Lobe	1	M
Intraventricular tumor	Frontal Lobe	8	M
Cavernous malformation	Temporal Lobe	11	F

**Table 2:** Simple Linear Regression Model for control and epileptic L2/3 PN HypO reversal potential.

	Equation	Slope, 95 % CI[]	Y-intercept, 95 % CI[]	R^2^
Control	Y = −0.406*X −28.06	−0.406, [−0.604, 0.208]	−28.06, [−41.08, −15.04]	0.847
Epilepsy	Y = −0.576*X −39.66	−0.576, [−0.950 to −0.202]	−39.66, [−64.24 to −15.07]	0.758

## Data Availability

Data will be made available on request.

## Appendix A. Supplementary data

Supplementary data to this article can be found online at https://doi.org/10.1016/j.nbd.2025.107252.
