# Supplementary material for: 4-Aminopyridine induced hyperpolarizing oscillations in pediatric human epileptic tissue are network-driven potassium currents that are abolished by activation of KCNQ2–5 (Kv7.2-Kv7.5) channels
Source: Neurobiol Dis. Author manuscript; Available in PMC 2026 Feb 15. (PMC12906913; doi:10.1016/j.nbd.2025.107252)
Supplement: Supplementary Data [file NIHMS2135426-supplement-Supplementary_Data.docx]

**Supplementary Data**

**
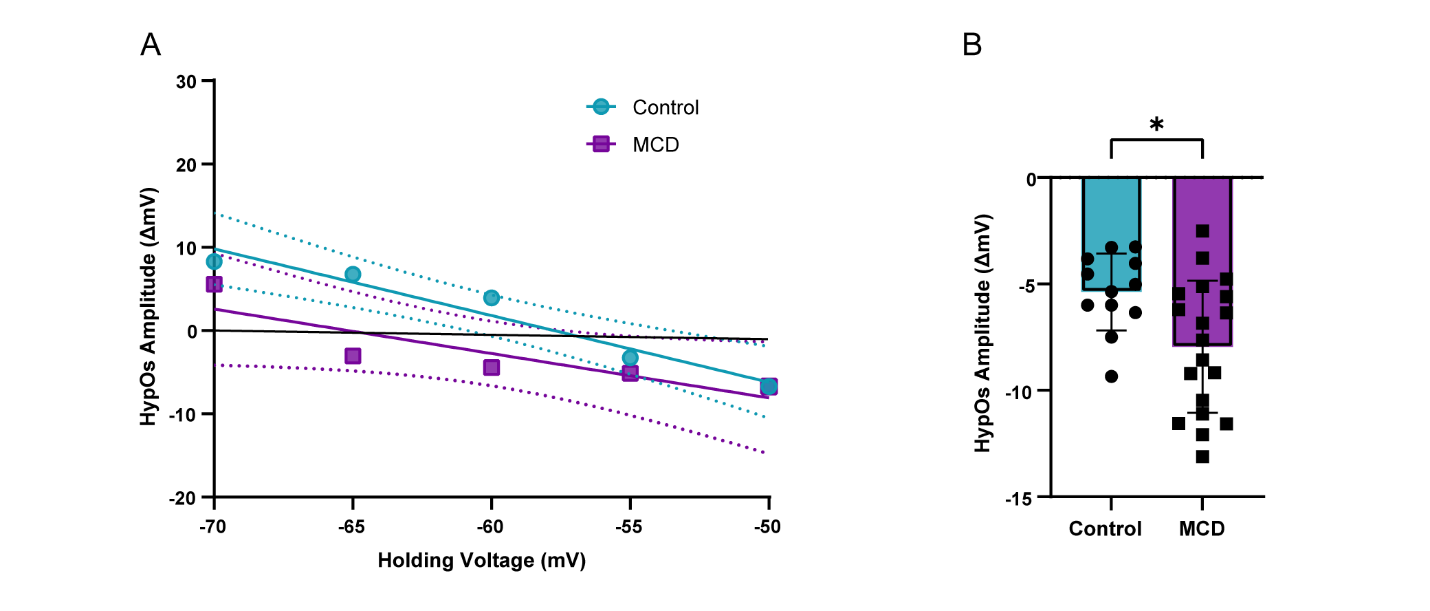
**

**Supplementary Figure 1. 4-AP-induced HypOs are elicited in mouse neocortical L2/3 PNs and are significantly larger in MCD mice compared to control.**

Average HypO amplitudes (ΔmV) at each holding voltage (mV) from control (GFP+) and MCD (Rheb^CA^-GFP+) L2/3 PNs with the corresponding linear regression line and 95% CI (control:-57.74mV (95% CI [-60.86 to -53.53],R^2^ = 0.95); MCD: -65.60mV (95% CI [-75.10 to -60.05], R^2^ = 0.83)) indicates a significant difference in MCD reversal potential y-interceptor elevation (F (1,8) = 11.27, *p* = 0.01). B) Control versus MCD HypO amplitudes (ΔmV) at -55mV indicate significantly larger HypOs in MCD mouse L2/3 PNs) (control: -5.37±1.81mV, n = 12, 6; MCD:-7.95 ± 3.10mV, n = 19, 6; unpaired t-test: *p* = 0.014).

**
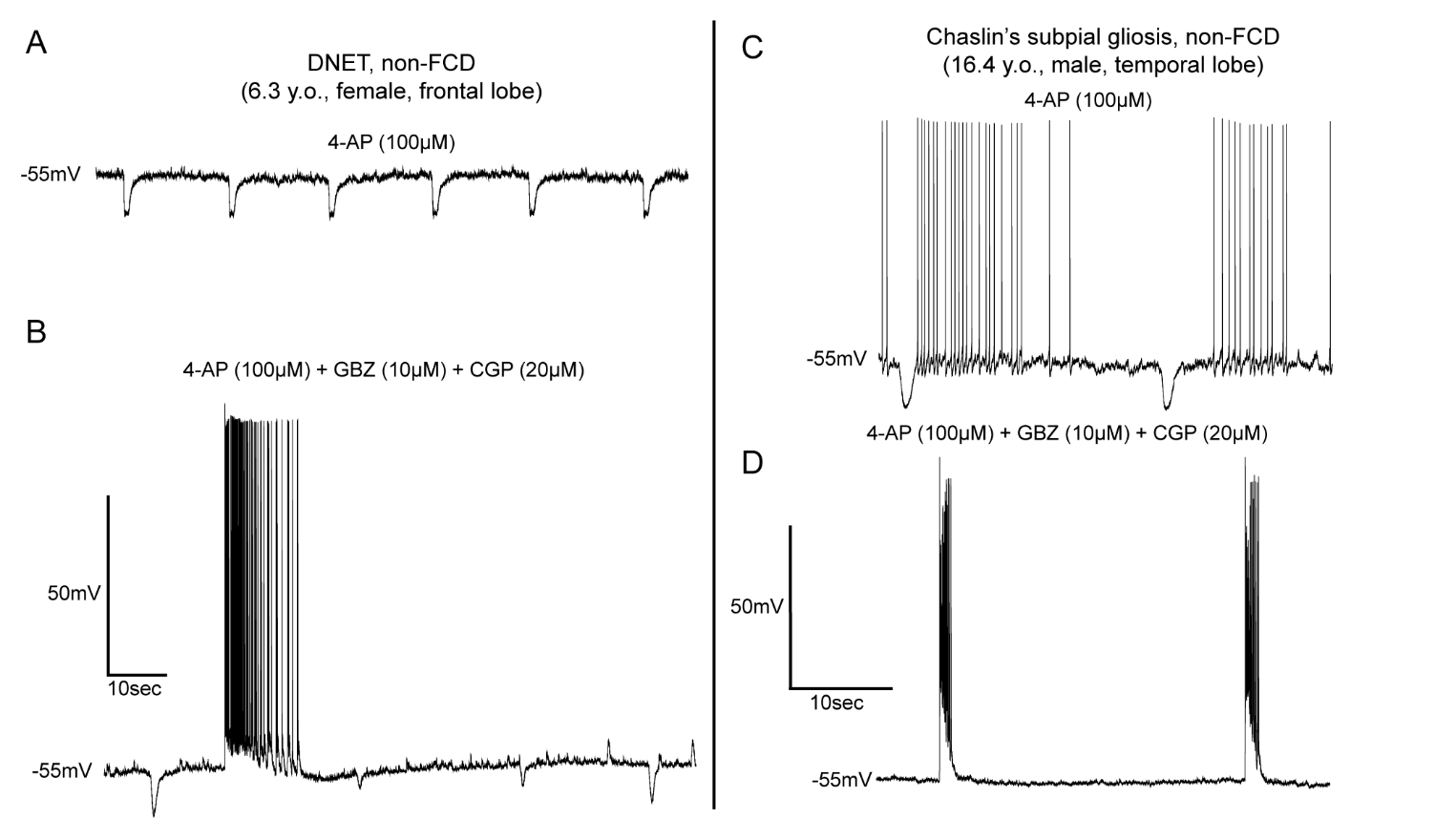
**

**Supplementary Figure 2:** GABAergic blockade causes a shift from 4-AP induced HypOs to neuronal bursting with HypOs (A-B) or neuronal bursting without HypOs (C-D).


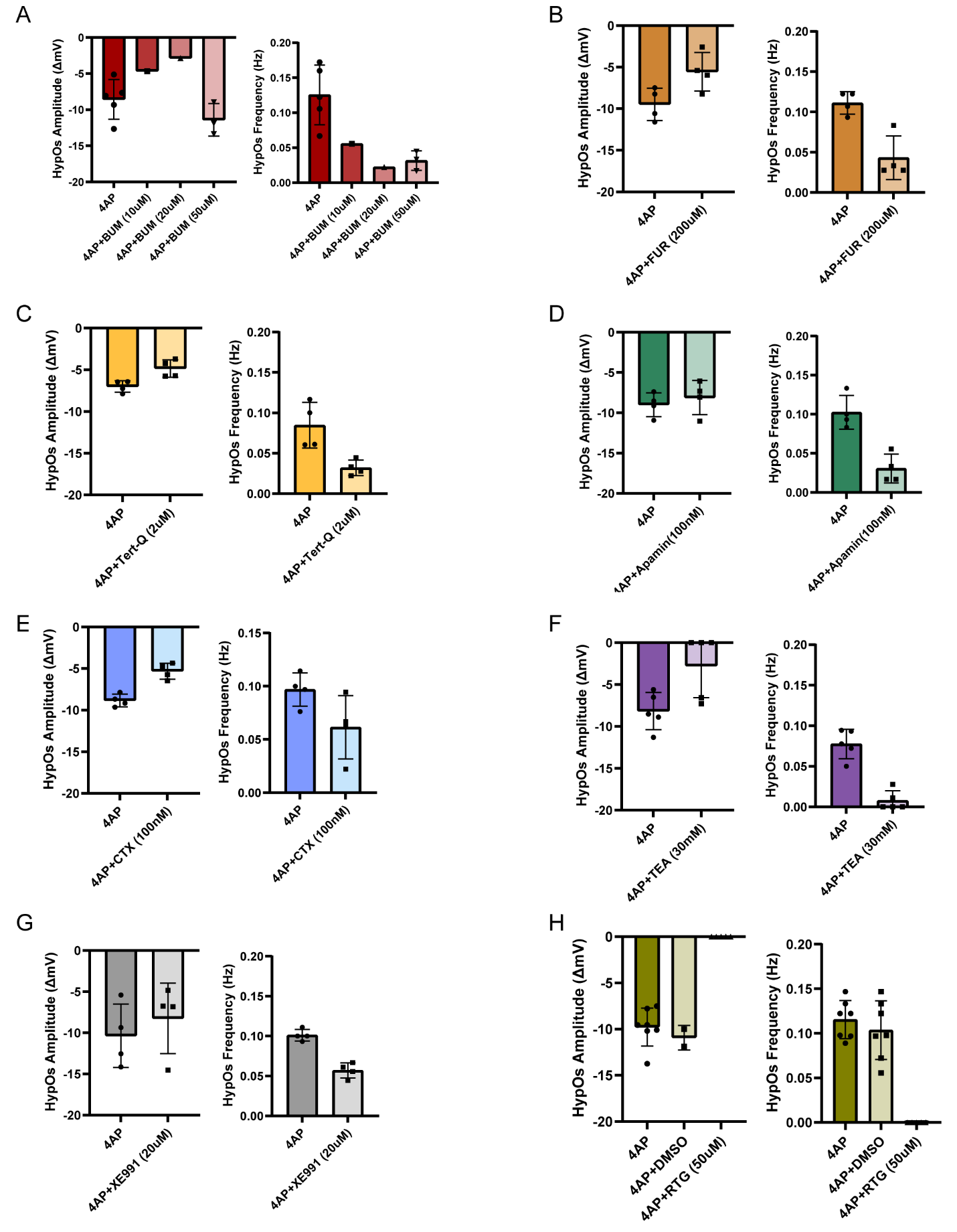


**Supplementary Figure 3. Unnormalized K^+^ channel blocker degree of blocking 4-AP-induced HypOs.** A-H, left) 4-AP (100μM) induced HypOs amplitude (mV) before and after drug wash on. A-H, right) 4-AP (100μM) induced HypOs frequency before and after drug wash on.

| Pathological Diagnosis | Location of Seizure focus (Lobe) | Seizure etiology | Seizure classification(s)  (Confirmed by EEG) | Anti-Seizure Medication(s) and Treatments  At Time of Surgery | Patient Age at Surgery | Age of Seizure Onset in Years | Patient Sex |
| --- | --- | --- | --- | --- | --- | --- | --- |
| Chaslin’s subpial gliosis | Temporal | Mesial Temporal Sclerosis | Focal onset with dyscognitive features | lamotrigine  zonisamade | 4 | 2 | F |
| Chaslin’s subpial gliosis (Prior resections consistent with Rasmussen) | Temporal frontal | Rasmussen Encephalitis | Focal aware | topiramate  clobazam | 16 | 5 | M |
| Chaslin’s subpial gliosis | Parietal | Perinatal stroke | 1. Focal sensory 2. Focal unaware 3. Focal to GTC | oxcarbazepine  Zonisamide | 11 | < 1 | M |
| Gliosis | Temporal | Perinatal stroke | 1. Epileptic spasms 2. Focal aware 3. Focal unaware | Clobazam  Valproic acid  Rufinamide  Modified Atkins Diet | 3 | < 1 | M |
| Gliosis | Temporal | Neonatal Intraventricular Hemorrhage | 1. Focal unaware 2. Tonic | Valproic acid  Lacosamide | 5 | < 1 | M |
| Low-grade glial neoplasm | Temporal | Temporal lesion | Focal motor/tonic | Levetiracetam  Topiramate | 1 | 1 | F |
| Low-grade glioma: WHO grade 1 | Temporal | Temporal lesion | 1. Focal unaware clonic 2. Focal unaware autonomic | Oxcarbazepine XR  Clobazam | 8 | 2 | M |
| DNET: WHO grade 1 | Frontal | Frontal lesion | 1. Focal unaware 2. Focal to GTC | Lacosamide  clobazam | 6 | 5 | F |
| FCD type IIa | Frontal | Fontal lesion | Focal tonic | Oxcarbazepine  Clobazam | 2 | 1 | F |
| FCD type IIa | Frontal | Fontal lesion | Focal unaware tonic | Oxcarbazepine  Clobazam  Lacosamide | 4 | 3 | M |
| Tuberous sclerosis | Temporal, parietal | Tuberous Sclerosis Complex | 1. Epileptic spasms 2. Atypical absence 3. Focal (electrographic only) | Clobazam | 2 | 2 | M |
| Chaslin’s subpial gliosis in cortex;  Hippocampal sclerosis | Temporal | Auto-inflammatory + structural lesion  (NORSE/ FIRES) | 1. Focal unaware autonomic 2. Focal aware sensory | Clobazam  Valproic Acid  Lacosamide  Anakinra | 15 | 11 | M |
| Gliosis | Temporal | Temporal lesion | 1. Focal unaware 2. Focal unaware to GTC | Valproic Acid  Clonazepam | 20 | 12 | M |
| Gliosis with inflammation | Temporal | PET lesion | Focal unaware sensory | Clobazam  Lacosamide  Zonisamide | 12 | 11 | M |
| Ganglioglioma: WHO grade 1 | Temporal | Temporal lesion | 1. Focal unaware 2. Focal to GTC 3. Focal aware | Levetiracetam  Carbamzaepine | 13 | 13 | F |
| Rasmussen Encephalitis | Temporal, frontal | Rasmussen Encephalitis | 1. Focal unaware 2. Epilepsia partialis continua 3. GTC 4. Subclinical | Levetiracetam  Clobazam | 4 | 3 | M |
| Hippocampal sclerosis | Temporal | Meningitis leading to Mesial Temporal Sclerosis | 1. Infantile spasms 2. Focal unaware | Levetiracetam  Lacosamide | 3 | < 1 | M |
| Ganglioglioma, WHO grade 1 | Temporal | Temporal lesion | Focal unaware | Carbamazepine  Zonisamide | 3 | 1 | M |
| FCD type IIa | Parietal | Parietal lesion | 1. Focal unaware 2. Focal aware clonic | Clobazam  Briviracetam | 5 | 1 | F |
| Chaslin’s subpial gliosis with hippocampal sclerosis | Temporal | Subdural Empyema leading to Mesial Temporal Sclerosis | 1. Focal unaware 2. GTC | Zonisamide  Medical marijuana  lacosamide | 12 | 9 | F |
| Diffuse low-grade glioma | Temporal | Temporal lesion | 1. Focal unaware 2. Infantile spasms 3. Generalized tonic | Vigabatrin  Levetiracetam | 1 | < 1 | F |
| Chaslin’s subpial gliosis | Temporal | Perinatal stroke | 1. Focal unaware 2. Focal tonic | Oxcarbazepine  Clobazam  Zonisamide  prednisolone | 4 | 1 | F |
| Chaslin’s subpial gliosis  (this was second surgery, likely MCD found in first case) | Occipital | Malformation of Cortical Development | 1. Focal unaware 2. Focal to generalized tonic (prior to first surgery) | Lamotrigine | 17 | < 1 | F |
| FCDIIID | Frontal, parietal | Perinatal stroke | 1. Focal unaware 2. Infantile spasms (prior to first surgery) | Lamotrigine | 12 | < 1 | M |
| Tuberous Sclerosis Complex | Frontal | Tuberous Sclerosis Complex | Focal unaware | Oxcarbazepine  Clobazam | 5 | < 1 | M |
| FCD type 1 | Temporal | Lesional (prior large tumor resected at age 3 months) | 1. Focal aware 2. Focal unaware +/- generalized clonic 3. Generalized status epilepticus | Oxcarbazepine  zonisamide | 7 | 1 | F |
| FCD type IIIB & DNET:  WHO grade 1 | Parietal | Parietal lesion | 1. Focal to GTC 2. Focal unaware | Lacosamide  Levetiracetam | 10 | 9 | F |
| FCD type 1A | Temporal | Temporal lesion | 1. Focal aware 2. Focal to GTC (prior to first surgery) | Lacosamide  Lamotrigine | 21 | 15 | F |
| Tuberous sclerosis | Occipital | Tuberous Sclerosis Complex | 1. Focal aware clonic 2. Focal to generalized tonic 3. Focal to generalized | Clobazam  CBD (Artisanal)  Zonisamide  Everolimus | 6 | < 1 | M |
| FCD type IIIc & Sturge-Weber Syndrome | Temporal | Sturge-Weber Syndrome | Focal aware | Oxcarbazepine  Levetiracetam  Zonisamde | 1 | < 1 | M |
| Gliosis (cortex)  Hippocampal Sclerosis | Temporal | Mesial temporal sclerosis + possible perinatal stroke | 1. Focal unaware 2. Focal to GTC 3. Focal aware | Oxcarbazepine XR  Lefetiracetam | 16 | 3 | F |
| Gliosis | Parietal | Presumed malformation of cortical development (not seen on path) | 1. Focal unaware 2. Focal to GTC | Lacosamide  Clobazam | 4 | 2 | F |
| Malformation of cortical development with oligodendroglial hyperplasia in frontal epilepsy (MOGHE) | Frontal | frontal lesion | 1. Tonic 2. Focal unaware | Valproic acid | 8 | 2 | M |
| Mild MCD | Temporal | KCNA2 mutation + temporal lesion | 1. GTC 2. Atypical absence | Lacosamide  Oxcarbazepine | 9 | < 1 | M |
| Gliosis | Temporal | Neonatal stroke | 1. Focal to GTC (requiring rescue med to abort) | Lamotrigine  Levetiracetam | 19 | 5 | M |
| Low-grade glioneuronal tumor | Temporal | Temporal lesion | 1. Focal unaware 2. Focal to generalized tonic (prior to first surgery) | Lacosamide | 6 | 6 | F |
| DNET: WHO grade 1 | Temporal | Temporal lesion | 1. Focal unaware | lacosamide | 16 | 9 | M |
| Tuberous Sclerosis | Frontal | Tuberous sclerosis complex | 1. Infantile spasms (only as infant) 2. Focal unaware | Zonisamide  Epidiolex | 4 | < 1 | F |
| Gliosis | Temporal | Temporal lesion | 1. Focal unaware | Lamotrigine  levetiracetam | 8 |  | F |

**Supplementary Table 1:** Patient Information including seizure onset, clinical seizure characteristics, and ASMs taken before resective surgery was performed.

| Drug | Final Concentration | Supplier | Catalog # |
| --- | --- | --- | --- |
| 4-aminopyridine | 100μM | Sigma-Aldrich | 275875 |
| Gabazine (GBZ) | 10μM | Abcam | ab144487 |
| CGP35348 (CGP) | 20μM | R&D | 123690-79-9 |
| Tetrodotoxin (TTX) | 1μM | Abcam | ab120055 |
| Bicuculline methiodide (BMI) | 20μM | Sigma-Aldrich | 14343 |
| Phaclofen (PH) | 20μM | Sigma-Aldrich | P118 |
| D-AP5 (AP-V) | 50μM | Abcam | ab120003 |
| DNQX | 20μM | Tocris | 0189 |
| Bumetanide (BUM) | 10, 20, 50μM | Sigma-Aldrich | B3023 |
| Furosemide (FUR) | 200μM | Sigma-Aldrich | F4381 |
| Tertiapin-Q (Tert-Q) | 2μM | Tocris | 1316 |
| Apamin | 100nM | Fisher Scientific | 50-197-0520 |
| Charybdotoxin (CTX) | 100nM | Fisher Scientific | 50-208-3025 |
| Tetraethylammonium (TEA) | 30mM | Sigma-Aldrich | T2265 |
| XE991 | 20μM | Tocris | 2000 |
| Retigabine (RTG) | 50μM | Sigma-Aldrich | SML0325 |
| CdCl_2_ | 200μM | Sigma-Aldrich | 202908 |
| BAPTA Tetrasodium Salt | 5-20mM | Sigma-Aldrich | 196418 |
| Niflumic Acid (NFA) | 300μM | Tocris | 4112 |
| T16Ainh-A01 | 25μM | Tocris | 4538 |
| Meclofenamic Acid (MFA) | 100μM | Fisher Scientific | AAJ6048403 |
| Carbenoxolone (CBX) | 300μM | Fisher Scientific | AAJ6371403 |
| Mefloquine hydrochloride (MFLQ) | 25μM | Sigma-Aldrich | M2319 |

**Supplementary Table 2**. Pharmacological manipulations used and the corresponding supplier and catalog number.

|  | Equation | Slope, 95% CI | Y-intercept, 95% CI | R^2^ |
| --- | --- | --- | --- | --- |
| Control  Mice | Y = -0.80*X – 46.24 | [-1.15, -0.45] | [-67.47, -25.00] | 0.95 |
| MCD  Mice | Y = -0.50*X – 33.24 | [-0.82 to -0.19] | [-53.19 to -13.29] | 0.83 |

**Supplementary Table 3.** Linear regression model for control and MCD mouse L2/3 PN HypO reversal potential
